# Interaction between row‐type genes in barley controls meristem determinacy and reveals novel routes to improved grain

**DOI:** 10.1111/nph.15548

**Published:** 2018-11-27

**Authors:** Monika Zwirek, Robbie Waugh, Sarah M. McKim

**Affiliations:** ^1^ Cell and Molecular Sciences The James Hutton Institute Invergowrie Dundee DD2 5DA UK; ^2^ Division of Plant Sciences University of Dundee at The James Hutton Institute Invergowrie Dundee DD2 5DA UK

**Keywords:** barley (*Hordeum vulgare*), branching, grain number, meristem, spikelet fertility, yield potential

## Abstract

- *Hordeum* species develop a central spikelet flanked by two lateral spikelets at each inflorescence node. In ‘two‐rowed’ spikes, the central spikelet alone is fertile and sets grain, while in ‘six‐rowed’ spikes, lateral spikelets can also produce grain. Induced loss‐of‐function alleles of any of five *Six‐rowed spike* (*VRS*) genes (*VRS1‐5*) cause complete to intermediate gains of lateral spikelet fertility. Current six‐row cultivars contain natural defective *vrs1* and *vrs5* alleles. Little information is known about *VRS* mechanism(s).
- We used comparative developmental, expression and genetic analyses on single and double *vrs* mutants to learn more about how *VRS* genes control development and assess their agronomic potential.
- We show that all *VRS* genes repress fertility at carpel and awn emergence in developing lateral spikelets. VRS4, VRS3 and VRS5 work through *VRS1* to suppress fertility, probably by inducing *VRS1* expression. Pairing *vrs3*,* vrs4* or *vrs5* alleles increased lateral spikelet fertility, despite the presence of a functional *VRS1* allele. The *vrs3* allele caused loss of spikelet identity and determinacy, improved grain homogeneity and increased tillering in a *vrs4* background, while with *vrs5*, decreased tiller number and increased grain weight.
- Interactions amongst *VRS* genes control spikelet infertility, determinacy and outgrowth, and novel routes to improving six‐row grain.

## Introduction

Grass inflorescence architecture is defined by the number, nature and arrangement of spikelets, the basic flowering unit, along single or branched reproductive axes (Kellogg, 2015). Spikelet number, fertility and survival determine grain yield, all of which are influenced by competition for finite resources. The *Triticeae* cereals usually form compact ‘spike’ inflorescences, characterised by sessile spikelets produced directly from alternating, opposing nodes on the branchless main axis or rachis. At each rachis node, *Hordeum* species develop a central spikelet flanked by two lateral spikelets. This distinctive triple spikelet arrangement arises when each inflorescence node forms a triple spikelet meristem (TSM) that cleaves into a large central spikelet meristem (CSM) bordered by two smaller lateral spikelet meristems (LSMs). Spikelet identity is marked by the initiation of two basal framing glumes, after which the remaining SM forms a single floret meristem (FM) that, if fertile, can develop a single grain. Lateral spikelet sterility is an ancestral trait of barley, *Hordeum vulgare* ssp. *spontaneum*, where the arrowhead shape formed by sterile lateral spikelets flanking a central grain is presumed to aid grain dispersal after rachis disarticulation (Harlan, 1968). However, during domestication, the selection of nonbrittle rachises supported the cultivation of variants with lateral spikelet fertility and six rows of grain on the spike (Helbaek, 1959; Harlan, 1968).

Despite their potential, cultivated six‐rowed barleys do not produce three‐fold more grain due to reduced tillering (basal branching) and rachis node number (Kirby & Riggs, 1978; Lundqvist *et al*., 1997). Moreover, lateral rows produce smaller and lighter grains compared with their central equivalents, making the size and weight of six‐rowed spike grain less uniform (Gupta *et al*., 2010; Lang *et al*., 2013). This is an undesirable trait for the premium end‐use malting sector, leaving six‐rowed varieties mostly grown for animal feed. Long‐standing interest in developing improved six‐row varieties has revealed that at least 11 loci regulate lateral spikelet fertility (Lundqvist & Lundqvist, 1988). To date, all alleles associated with lateral spikelet fertility are predicted to be impaired or loss‐of‐function alleles, many of which cause an intermediate phenotype between the two and six‐row form (Lundqvist & Lundqvist, 1988). Induced recessive alleles at five major row‐type loci can independently convert spikes from two‐rowed to six‐rowed: *SIX‐ROWED SPIKE 1 (VRS1)*,* VRS2*,* VRS3* (syn. *INTERMEDIUM‐A*), *VRS4* (syn. *INTERMEDIUM‐E*) and *VRS5* (syn. *INTERMEDIUM‐C*). Six‐rowed barley originated with multiple natural recessive *vrs1* alleles (Tanno *et al*., 2002), which are now accompanied by a natural allele of *vrs5* (*Int‐c.a*) that confers improved lateral grain fill in six‐row cultivars (Harlan, 1968; Lundqvist *et al*., 1997). The three other major recessive row‐type alleles are not prevalent in current six‐row cultivars (Koppolu *et al*., 2013; Bull *et al*., 2017; Youssef *et al*., 2017).

Recent efforts have revealed the molecular identity of the five major row‐type genes. *VRS5* encodes a class II TCP transcription factor whose homologues in maize, rice, wheat and Arabidopsis repress tiller bud outgrowth to promote apical dominance (Doebley *et al*., 1995, 1997; Takeda *et al*., 2003; Lewis *et al*., 2008; Ramsay *et al*., 2011; Dixon *et al*., 2018). *VRS1* encodes a homeodomain‐leucine zipper class I protein (HD‐ZIP1; Komatsuda *et al*., 2007) whose homologue in maize, *grassy tillers1* (*gt1*) inhibits tiller bud outgrowth downstream of *tb1* (Whipple *et al*., 2011). *VRS4* encodes a LATERAL ORGAN BOUNDARIES (LOB) transcription factor orthologous to the maize *ramosa2* gene that prevents ectopic branching in maize ears and tassels (Bortiri *et al*., 2006; Koppolu *et al*., 2013). In addition to controlling row‐type, *VRS4* promotes spikelet and floret determinacy (Koppolu *et al*., 2013). *VRS2* encodes a homologue of the Arabidopsis *SHORT INTERNODES* gene, whose loss of function is associated with hormonal imbalances between auxin and cytokinin along the spike (Youssef *et al*., 2017). *VRS3* encodes a putative Jumonji C‐type (JMJC) H3K9me2/3 histone demethylase (Bull *et al*., 2017; van Esse *et al*., 2017), orthologous to the rice gene *OsJMJ706* that regulates rice spikelet morphology (Sun & Zhou, 2008). Comparative transcriptomics suggests that *VRS3* promotes the expression of other *VRS* genes (Bull *et al*., 2017; van Esse *et al*., 2017).

Understanding the molecular mechanisms underlying row‐type and its relationship to tillering and grain parameters may help to overcome the limitations of six‐rowed varieties. However, studies on row‐type are often confounded by the sensitivity of the phenotype to both genetic background and the environment. To address this, we assembled a panel of near‐isogenic *vrs1−vrs5* alleles in a common two‐row cultivar (cv) Bowman recurrent parent, and used crossing to generate a series of near‐isogenic double mutants. By comparative analyses in single and double mutants, we asked several questions:Does lateral spikelet fertility develop in a similar way in different *vrs* mutants? How does variation in row‐type genes influence each other's expression and function?What is the genetic hierarchy of *VRS* gene function in the control of spike architecture? Can novel *vrs* allelic combinations increase tillering and grain homogeneity?

## Materials and Methods

### Germplasm and growth conditions

Barley (*Hordeum vulgare* ssp*. vulgare)* near‐isogenic lines for *vrs1−vrs5* row‐type genes in cv Bowman (Druka *et al*., 2011) were obtained from The James Hutton Institute seed store. Allele designations, mutagen and molecular nature, backcross generation and common names used for clarity in this manuscript are given in Table 1. Double mutants *vrs1vrs3*,* vrs1vrs4*,* vrs1vrs5*,* vrs3vrs4*,* vrs3*,*vrs5*, and *vrs4vrs5* were generated by crossing the relevant nearly isogenic lines and selecting homozygous progenies using allele‐specific molecular diagnostics (Supporting Information Method S1). Plants were grown in glasshouses under long‐day conditions (light : dark, 16 h : 8 h, 18°C : 14°C) in 15 cm (crossing and phenotyping) and 7 cm (microscopy and molecular analyses) diameter pots filled with a standard cereal compost mix.

**Table 1:** Characteristics and nomenclature of alleles used throughout this article

Allele	Mutagen	Molecular nature	Bowman near‐isogenic line (backcross generation)	Names used here	Comments
*vrs1.a*	natural	1 bp deletion in exon 2 leading to frameshift (E152<)^1^	BW898 (BC=<7S3)	*vrs1*	common 6‐row allele
*vrs1.c*	natural	unknown	BW899 (BC6S3)	*vrs1.c*	Awnless/awnletted fertile lateral spikelets^2^
*vrs2.e*	X‐ray	1 bp deletion leading to frameshift (C18<)^3^	BW901 (BC5S3)	*vrs2*	limited fertility in basal lateral spikelets
*int‐a.1*	X‐ray	2 bp deletion leading to frameshift (R529<)^4^^,^ 5	BW419 (BC6S3)	*vrs3*	weak *vrs3* allele
*vrs3.f*	X‐ray	1 bp deletion leading to frameshift (C133)^4^^,^ 5	BW902 (BC6S3)	*vrs3.f*	strong *vrs3* allele
*int‐e.58*	EMS	NS SNP in the coding region for LOB‐domain leading to A83V^6^	BW423 (BC6S3)	*vrs4*	weak *vrs4* allele
*vrs4.k*	Gamma ray	1 bp deletion leading to frameshift (E17<)^6^	BW903 (BC6S3)	*vrs4.k*	strong *vrs4* allele
*int‐c.5*	X‐ray	1 bp deletion causing frameshift (T279<)^7^	BW421 (BC6S3)	*vrs5*	recessive

Alleles are in *Hordeum vulgare* ssp. *vulgare*. EMS, ethyl methanesulfonate; NS, nonsynonymous; SNP, single nucleotide polymorphism. ^1^Komatsuda *et al*., 2007; ^2^Konishi & Franckowiak, 1997; ^3^Youssef *et al*., 2017; ^4^Bull *et al*., 2017; ^5^van Esse *et al*., 2017; ^6^Koppolu *et al*., 2013; ^7^Ramsay *et al*., 2011.

### Scanning electron microscopy

Main culm spikes harvested from plants between 9 and 28 d after germination (dag) were processed as described (Houston *et al*., 2013) and imaged using an environmental scanning electron microscope under a 10–15 kV acceleration voltage.

### Phenotyping

Spikelet fertility of the main culm spike per individual was scored using grain and awn formation as a proxy at a whole spike scale for *n* = 10 spikes/genotype and along each rachis node for *n* = 5 spikes/genotype. The numbers of rachis nodes, and any additional branches and/or spikelets on the main spike were recorded. Tiller number was recorded at 5 d after germination (dag) and repeated at 5 d intervals until plants ceased to produce new tillers (*n* = 6–14 individuals/genotype). Tiller number included tillers with visible spikes as well as ‘vegetative tillers’ lacking visible spikes. Grain width, length, area and thousand grain weight (TGW), along with grain number per spike, were measured using a Marvin Grain Analyser (GTA Sensorik, Neubrandenburg, Germany) from grain harvested from the main culm and tallest tiller per individual (*n* = 10 individuals/genotype). Heading date was defined as the emergence of the main culm spike at 5 cm above the sheath of the upper leaf (Zadoks stage 55; Zadoks *et al*., 1974). Following Shapiro−Wilk tests for normality, pairwise comparisons were analysed with two‐tailed Student's *t*‐test and multiple comparisons with one‐way analysis of variance (ANOVA) combined with Tukey's test.

### RNA extraction and cDNA synthesis

Total RNA was extracted from five to seven main culm spikes per biological replicate (*n* = 3 replicates/genotype) at double ridge (DR)/TSM, glume primordium (GP)/lemma primordium (LP), stamen primordium (SP)/awn primordium (AP) and white anther (WA) stages (according to Kirby and Appleyard, 1984) from Bowman and single *vrs* mutants (Fig. 2) and at GP, LP, SP and AP stages for double mutants, parents and Bowman (Fig. 5), using the RNeasy Plant Mini Kit (Qiagen) followed by on‐column DNA removal using a RNase‐Free DNase set (Qiagen). RNA integrity was determined by gel electrophoresis. cDNA was synthesised from 1 μg of RNA with oligo(dT)_23_VN primers (New England Biolabs (NEB), Ipswich, MA, USA) using ProtoScript^®^ II Reverse Transcriptase (NEB).

### Quantitative real‐time polymerase chain reaction (qRT‐PCR)

qRT‐PCRs were conducted according to Bustin *et al*. (2009), with primers, primer efficiency and *R* ^2^, as described in Method S1. Expression was considered differential if *P‐*values were < 0.05 with Student's *t*‐test (two‐tailed) and > 50% altered. Bar graphs represent mean normalised expression (mne; Simon, 2003).

## Results

### Variation in spike morphology across a row‐type near‐isogenic line panel

**Figure 1:**
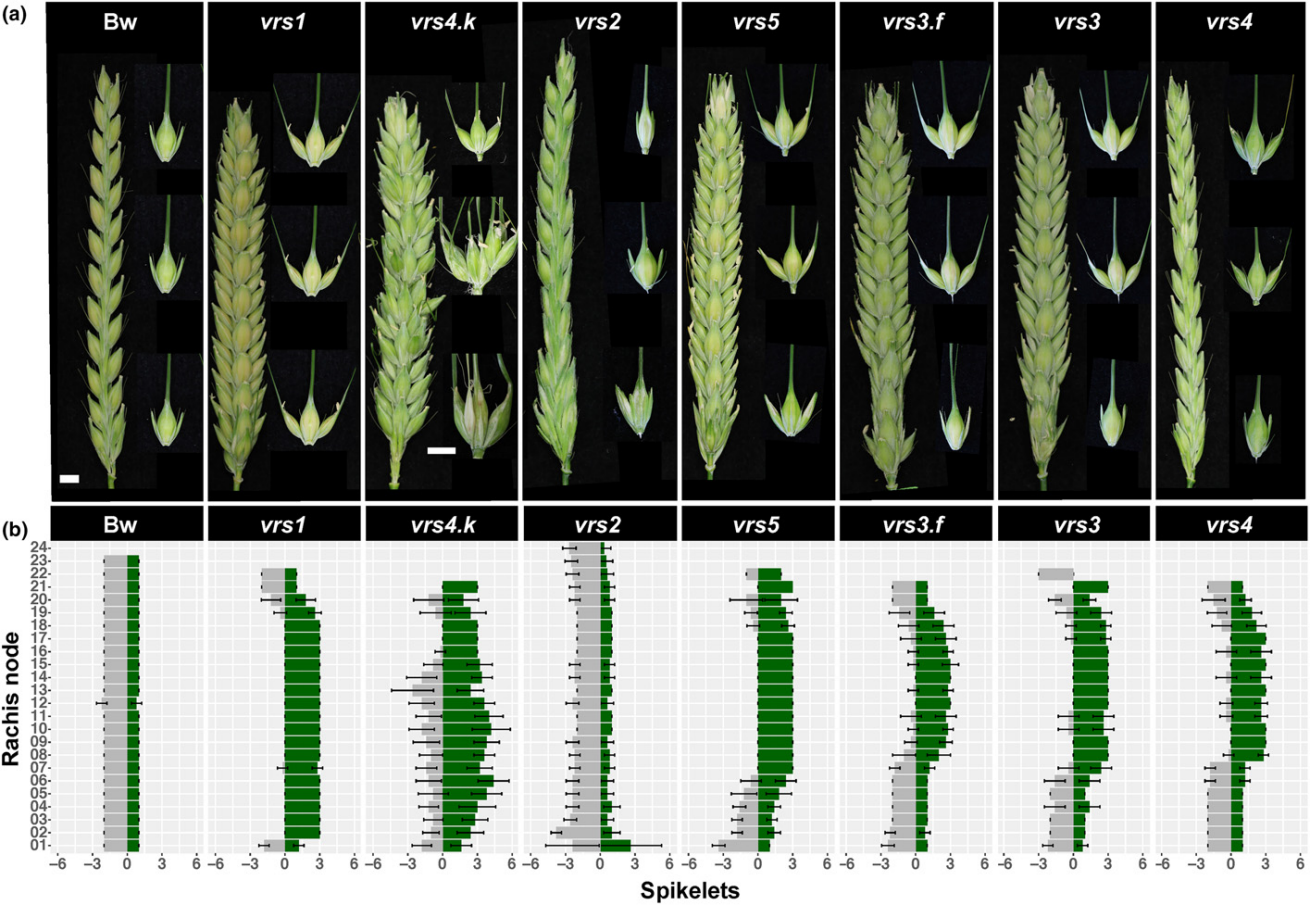
*Hordeum vulgare* ssp*. vulgare* single *vrs* mutants show gradients in lateral spikelet fertility, awn development and additional spikelet formation along the rachis. (a) Spikes of Bowman (Bw) and *vrs1*,* vrs4.k*,* vrs2*,* vrs5*,* vrs3.f*,* vrs3* and *vrs4* mutants. Awns were removed from whole spikes for clarity. Representative spikelets from basal, central and apical nodes are displayed to the right of each spike. The scale bar in Bw (a) applies to all, except *vrs4.k* middle and basal spikelets for which the scale bar underneath *vrs4.k* basal spikelet applies. An asterisk indicates the small awn of the *vrs5* lateral spikelet. Bar, 0.5 cm. (b) Distribution of grain‐setting fertile (green bars), and empty (grey bars) spikelets at individual rachis nodes along the main culm spike rachis. Bars represent mean values (± SD) calculated from main culm spikes (*n* = 5).

To compare *vrs* spike morphology directly, we grew and phenotyped Bowman and a set of Bowman near‐isogenic lines, each containing one of the five major recessive *vrs* alleles (Druka *et al*., 2011; Table 1). We used the induced *int‐c.5* allele, not the natural *Int‐c.a* allele for two reasons: (1) *int‐c.5* and not *Int‐c.a* had been introgressed into Bowman; and (2) we wanted to explore *int‐c.5*'s genetic potential. We measured spikelet traits from 10 spikes/genotype (combined spikelets, Table 2; central, lateral and additional spikelets separated, Table S1) and trends along the rachis of five spikes/genotype (Fig. 1a,b; Table S2). As expected, Bowman lateral spikelets were rudimentary and infertile, such that each node presented a single grain derived from the central spikelet (Fig. 1a,b; Table 2). All row‐type alleles increased lateral spikelet development with distinct trends along the rachis (Fig. 1a,b; Tables S1, S2) that altered grain set per node (Table 2): *vrs1* and the strong *vrs4.k* allele spikes showed the most robust lateral spikelet fertility, only lacking grain in lateral spikelets at the top and basal nodes, and increased grain set per node to 2.6 (± 0.2) and 2.9 (± 0.2) respectively; *vrs2* spikes showed extensive sterility (0.6 ± 0.1 grain per node) and increased development of lateral spikelets only at basal nodes; while *vrs3*,* vrs3.f* (strong allele), *vrs4* and *vrs5* mutants showed lateral spikelet fertility in the middle to upper spike, leading to increases in grain set per node of between 1.6 (± 0.3) and 2.0 (± 0.9). While Bowman lateral spikelets were awnless, row‐type mutants’ lateral spikelets setting grain always developed awns (long needle‐like projections) off the lemma (awned spikes in Fig. S1), as reported in other cultivars (Gustafsson & Lundqvist, 1980; Lundqvist & Lundqvist, 1988), although awns on *vrs5* lateral spikelets were small (< 2 cm long; Figs 1a, S1). However, many lateral spikelets were both empty and awned in *vrs3* and *vrs4* (Table S1). All row‐type mutants, except *vrs2*, also had a reduced number of rachis nodes (Fig. 1b; Table 2).

**Table 2:** Spike parameters of Bowman, single *vrs* mutant and double *vrs* mutants

Line	Rachis nodes	Spikelets/spike (/node)	Spikelets setting grain/spike (/node)	Empty awned spikelets/spike (/node)	Grain area (mm^2^)^	TGW (g)
Bw	22.4 ± 1.3	66.9 ± 3.4 (3.0 ± 0.0)	19.1 ± 2.5 (0.9 ± 0.1)	3.0 ± 1.5 (0.1 ± 0.1)	27.2 ± 1.5	56.5 ± 4.6
*vrs1*	20.4 ± 0.5***	60.3 ± 2.1* (3.0 ± 0.1)	52.7 ± 4.2*** (2.6 ± 0.2***)	7.7 ± 4.2*** (0.4 ± 0.2***)	22.2 ± 3.1 ***	37.7 ± 11 ***
*vrs2*	**23.6 ± 0.5**	73.6 ± 3.1* (3.1 ± 0.2)	14.6 ± 2.9* (0.6 ± 0.1*)	20 ± 1.4 d (0.8 ± 0.0***)	24.2 ± 3.1**	48.1 ± 7.6 ***
*vrs3.f*	20.6 ± 0.5 a	62.5 ± 1.6** (3.0 ± 0.1)	35.8 ± 7.4*** (1.7 ± 0.4***)	8.75 ± 4.3** (0.4 ± 0.2***)	**23.2 ± 4.1**	39.6 ± 13 *
*vrs3*	19.5 ± 1.2***	58.5 ± 3.7** (3.0 ± 0.0)	31.2 ± 4.7*** (1.6 ± 0.3***)	13.5 ± 2.1*** (0.7 ± 0.1***)	22.6 ± 0.9***	36.8 ± 7.1***
*vrs4.k*	19.6 ± 1.1***	78.4 ± 6.6*** (4.0 ± 0.3***)	56.4 ± 4.9*** (2.9 ± 0.2***)	13.4 ± 5.0*** (0.7 ± 0.3***)	20.7 ± 1.7***	34.9 ± 6.5***
*vrs4*	19.5 ± 1.2***	58.5 ± 3.7** (3.0 ± 0.0)	37.3 ± 4.8*** (2.0 ± 0.9***)	19.5 ± 6.1*** (0.9 ± 0.3***)	22.2 ± 0.5***	38.2 ± 2.3***
*vrs5*	19.5 ± 1.6***	58.5 ± 4.9** (3.0 ± 0.1)	34.5 ± 3.2*** (1.8 ± 0.3***)	8.5 ± 4** (0.4 ± 0.2***)	22.3 ± 4.4***	43.1 ± 16***
*vrs1vrs3*	**22.4 ± 2.7**	70.8 ± 7.7 (3.1 ± 0.0)	56.4 ± 9.5*** (2.5 ± 0.6***)	11.0 ± 8.2* (0.5 ± 0.3*)	19.83 ± 1.5***	20.7 ± 3.2***
*vrs1vrs4*	20.4 ± 0.4***	61.2 ± 2.5* (3.0 ± 0.1)	49.4 ± 5.3*** (2.4 ± 0.2***)	9.3 ± 3.3*** (0.5 ± 0.2***)	18.1 ± 1.1***	24.2 ± 5.8***
*vrs1vrs5*	18.2 ± 1.3***	57.4 ± 5.5* (3.2 ± 0.6)	48.4 ± 7.8*** (2.7 ± 0.5***)	3.20 ± 2.8 **(0.2 ± 0.1)**	25.3 ± 1.42*	49 ± 4.5**
*vrs3vrs4*	**21.6 ± 2.7**	84 ± 8.6*** (3.9 ± 0.5***)	43.8 ± 14.9*** (2.1 ± 0.8***)	35.8 ± 16** (1.7 ± 0.7***)	21.4 ± 1.3***	41.2 ± 2.4***
*vrs3vrs5*	17.4 ± 1.3***	59.7 ± 4.5** (3.4 ± 0.2)	41.4 ± 2.6*** (2.4 ± 0.2***)	14.7 ± 4.1*** (0.8 ± 0.2***)	**28.1 ± 1.4**	**54.1 ± 2.3**
*vrs4vrs5*	17.4 ± 1.4***	53.4 ± 4.1* (3.1 ± 0.1)	46.7 ± 5.6*** (2.7 ± 0.2***)	**3.8 ± 2.6**(0.2 ± 0.2)	24.9 ± 0.9***	46.1 ± 4.5***

Spike parameters of *Hordeum vulgare* ssp. *vulgare* single *vrs* mutants. Values are mean (± SD) from awned spikelets from *n* = 10 spikes per line. ^Averaged values for grains deriving from central, lateral and additional spikelets. Significant differences between Bowman and mutants indicated by *, *P *<* *0.05; **, *P *<* *0.01; ***, *P *<* *0.001 (Student's *t*‐test). Bold text indicates no significant difference to Bowman. Bw, Bowman; TGW, thousand grain weight.

Consistent with Koppolu *et al*. (2013), *vrs4.k* spikes had 19.5 (± 5.6) fertile, awned, additional spikelets leading to an average of 4.0 (± 0.3) spikelets per node (Table 2), and occasionally developed short ectopic spike branches from middle nodes (Fig. 1a,b). The *vrs2* spikes developed 2.8 (± 1.5) rudimentary, awnless additional spikelets in each of the two basal nodes (Fig. 1a,b; Tables S1, S2), similar to that found by Youssef *et al*. (2017). While, rarely, the middle nodes of *vrs3.f* developed one and the basal rachis nodes above the collar in *vrs5* developed one to three awnless, infertile additional spikelets (Tables S1, S2).

### Gain of rapid, coordinated emergence of carpel and awn primordia is associated with lateral spikelet fertility

**Figure 2:**
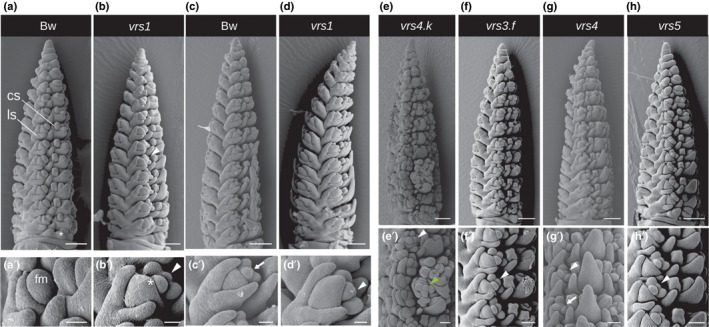
*Hordeum vulgare* ssp. *vulgare* single *vrs* mutants and Bowman (Bw) lateral spikelet ontogeny. Scanning electron microscopy of spikes at (a, b, e–h) early awn primordium (AP) and (c, d) later AP stage. (a′–h′) Close‐up images of lateral spikelets. (b′, d′, h′) White arrowheads indicate carpel primordia in lateral florets. (e′) Green arrowhead shows carpel primordium in *vrs4.k* additional spikelet. (c′, g′) Arrow shows the absence of carpel primordium in lateral florets; *, awn primordium. Bars: (a–h), 250 μm; (a′–h′), 50 μm. Staging according to Kirby & Appleyard (1984). cs, central spikelet; ls, lateral spikelet; fm, floret meristem.

We first explored whether lateral spikelet fertility across the panel initiated in the same way. Clear differences emerged by the early AP stage. Bowman lateral spikelets were small with short glumes, had limited lemma growth and no awn differentiation (Fig. 2a, a′). By contrast, *vrs1* lateral spikelets were larger with well developed glumes and enlarged lemmas that were hooded with triangular awn primordia (Fig. 2b,b′). Similar to findings for central spikelets, *vrs1* lateral spikelets had already initiated stamen and carpel primordia, while Bowman only initiated stamen primordia in late AP stage without accompanying carpel or awn primordia (Fig. 2c,c′), by which time *vrs1* awns enclosed the developing floret organs (Fig. 2d,d′). Carpel and awn primordia in *vrs4.k* developed on both lateral and additional spikelets (Fig. 2e,e′). Lateral spikelets in *vrs3.f*,* vrs4 and vrs5* also initiated carpel primordia and awns at the AP stage, except at some basal nodes, with more variable awn growth than in *vrs1* (Figs 2f–h,f′–h′, S2). *vrs5* uniquely showed butterfly‐shaped anther lobes in basal lateral spikelets compared with the less‐developed stamen primordia in Bowman and other row‐type alleles (Fig. S2). Therefore, lemma growth, awn initiation and carpel primordia emergence represent a suite of a morphological events normally repressed by row‐type gene function.

### *VRS* gene expression is dependent on *VRS* gene function

**Figure 3:**
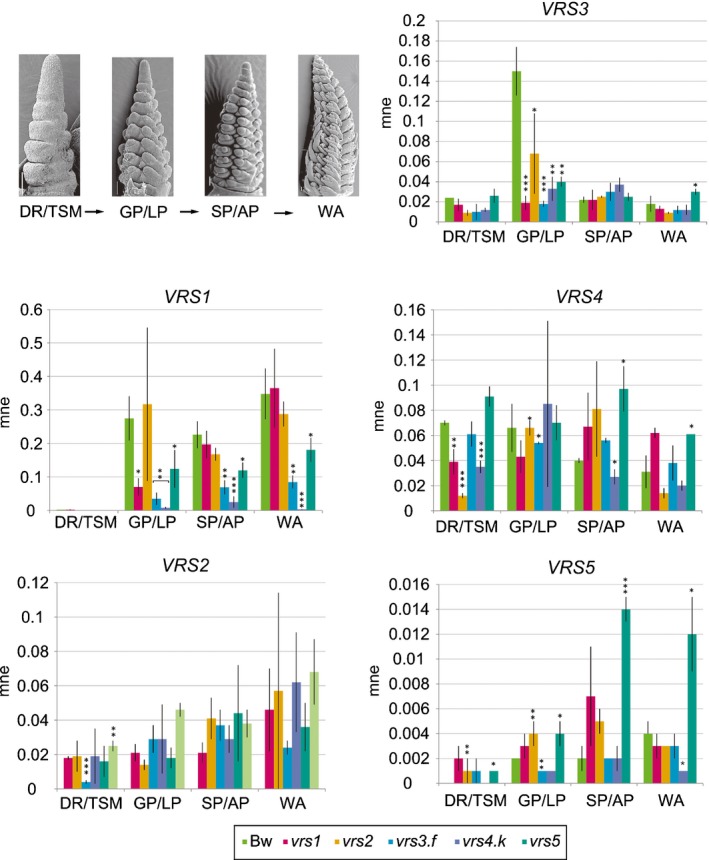
*VRS* gene expression in *Hordeum vulgare* ssp. *vulgare* single *vrs* mutants and Bowman (Bw) during spike development. Scanning electron micrographs in the top left panel show Bowman spikes at the double ridge/triple spikelet mersitem (DR/TSM), glume primordium/lemma primordium (GP/LP) and stamen primordium/awn primordium (SP/AP) and white anther (WA) stages. Bar graphs show the mean normalised expression (mne) (± SD) of *VRS1‐5* genes determined by qPCR on DR/TSM, GP/LP, SP or WA‐staged spikes (*n* = 3). Significant differences to Bowman indicated by *, *P* < 0 0.05; **, *P* < 0 0.01; ***, *P* < 0.001 (Student's *t*‐test).

To establish how *VRS* gene expression is related to lateral spikelet in/fertility, we constructed a gene expression by genotype profile by developmental stage matrix (Fig. 3; plotted values and statistics in Table S3). In Bowman, *VRS1* was highly expressed at GP/LP, SP/AP and WA. *VRS1* expression was reduced in *vrs4.k* (by 97% at GP/LP, 89% at SP/AP, and 99% at WA, *P* < 0.005), and in *vrs3.f* (87% at GP/LP, 69% at SP/AP, and 76% at WA, *P* < 0.005), showing that VRS4, and to a lesser extent VRS3, function is important to induce *VRS1*. However, *VRS1* levels were only 55% reduced at GP/LP, and 47% at SP/AP and WA (*P* < 0.05) in *vrs5*, suggesting that VRS5 contributes less to *VRS1* expression compared with VRS3 and VRS4.

Consistent with inducing *VRS1* expression, *VRS4* levels were highest at DR/TSM and *VRS3* at GP/LP. *VRS5* transcripts were lowly expressed at all stages and *VRS2* expressed most highly at WA. *VRS3* expression was specifically reduced at GP/LP across all lines, suggesting that *VRS3* expression is sensitive to the row‐type switch, potentially in a feed‐forward loop dependent on *VRS1* activation. By contrast, *VRS4* expression is only reduced in *vrs1* and *vrs2* at DR/TSM, although as this precedes significant *VRS1* and *VRS2* expression, this may be a pleiotropic effect. Rather, *VRS4* transcript abundance increased later on, especially in *vrs5* (117% at SP/AP, *P* < 0.05; 400% at WA), suggesting that VRS5 may be important to fine tune *VRS4* expression during spikelet differentiation. *VRS5* levels were lower in *vrs3.f* at GP/LP (50%, *P* < 0.05), and WA in *vrs4.k* (75%, *P* < 0.05). *VRS2* expression was little altered across the lines.

### Genetic analyses of double *vrs* mutants

**Figure 4:**
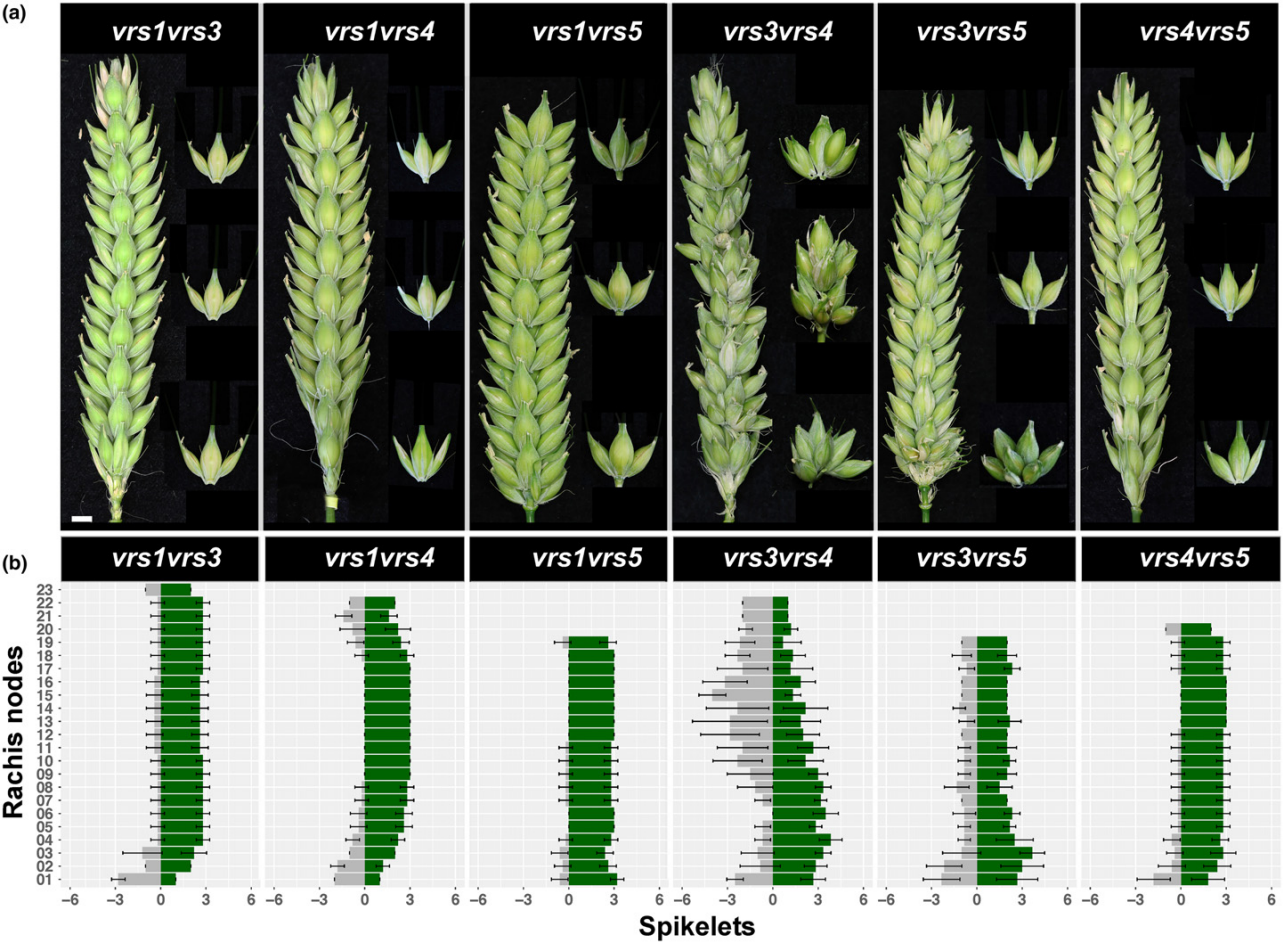
*Hordeum vulgare* ssp. *vulgare* double *vrs* mutants show novel routes to six rows. (a) Spikes of *vrs1vrs3*,* vrs1vrs4*,* vrs1vrs5*,* vrs3vrs4*,* vrs3vrs5*, and *vrs4vrs5*. The awns were removed from the whole spikes for clarity. Representative spikelets from basal, central and apical nodes are displayed to the right. The middle node of *vrs3vrs4* contains a spike branch. Scale bar in *vrs1vrs3* applies to all images. Bar, 0.5 cm. (b) Distribution of grain‐setting fertile (green bars), and infertile (grey bars) spikelets at individual rachis nodes along the main culm spike rachis. Bars represent mean values (± SD) calculated from the main culm spikes (*n* = 5).

To test regulatory associations amongst *VRS* genes in the control of row‐type and assess the potential of novel *vrs* combinations, we generated six double mutants: *vrs1vrs3*,* vrs1vrs4*,* vrs1vrs5*,* vrs3vrs4*,* vrs3vrs5*, and *vrs4vrs5*; and phenotyped as before, comparing with Bowman (Figs 4, S3; Tables 2, S1, S2) and comparing with parents (Table S4). Gains in *vrs1vrs3*,* vrs1vrs4* and *vrs1vrs5* lateral spikelet fertility increased grain number per spike greater than the *vrs3/4/5* parents and equal those in *vrs1* (Tables S1, S4), suggesting that *VRS3*,* VRS4* and *VRS5* work through *VRS1* to control row‐type. However, grain distribution was noticeably more even along *vrs1vrs5* spikes (Figs 1, 4), suggesting a more complete gain of lateral fertility. Strikingly, double *vrs3*,* 4* and *5* mutants showed an increased level of lateral spikelet fertility similar to *vrs1*, despite containing the functional *Vrs1* allele. *vrs4vrs5* spikes increased grain number and showed a gain of lateral fertility in basal nodes compared with their parents, while *vrs3vrs4* and *vrs3vrs5* double mutants also generated awned lateral spikelets throughout their spikes (Figs 4, S3; Tables S1, S2, S4), although many of these spikelets were empty (Table S1). Rachis node number increased in *vrs1vrs3* and *vrs3vrs4* to be equivalent to Bowman, in contrast with other double mutants (Tables 2, S4), removing the rachis node penalty associated with lateral spikelet fertility.

### *VRS3* promotes spikelet identity and determinacy

**Figure 5:**
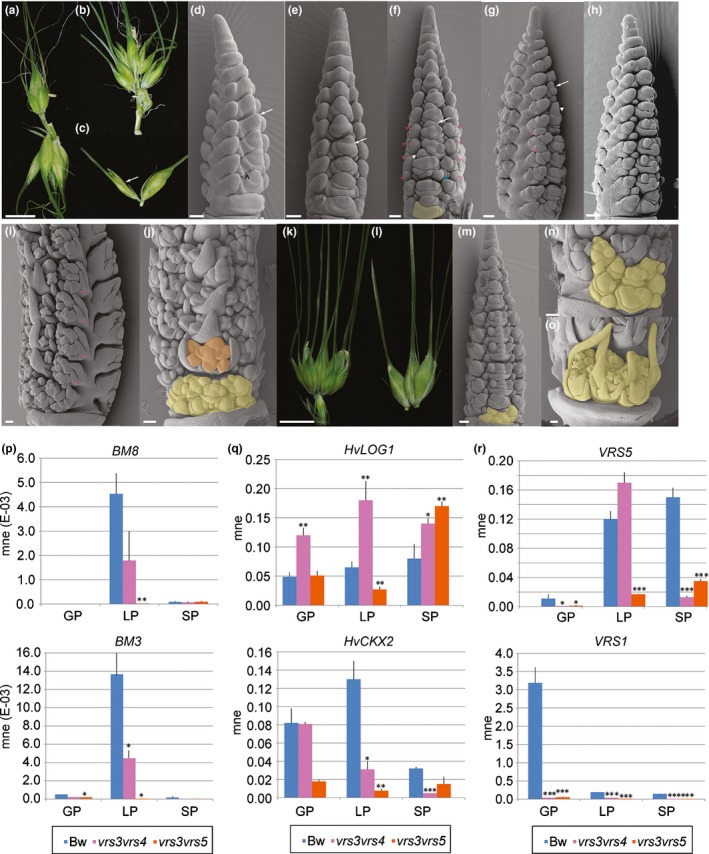
Defective *VRS3* combined with either impaired *VRS4* or *VRS5* alleles in *Hordeum vulgare* ssp. *vulgare* leads to aberrant meristem identity and determinacy. (a–g) *vrs3vrs4*: (a) extended rachilla bearing a triplet; (b) multi‐noded branch from middle spike node; (c) glume to spikelet conversion (arrow); (d, e) scanning electron micrographs (SEMs) of spikes at lemma primordium (LP) stage. (f, g) SEM at stamen primordium (SP) stage. (h) SEM of Bowman (Bw) at SP stage. (i–j) SEM of *vrs3vrs4* late awn primordium (AP) stage. ^ in (d) shows the central glume axillary meristem. White arrows in (d–g) show ectopic spike branch meristem formation, pink arrowheads show additional spikelet meristems formed adjacent to/in place of lateral spikelet glumes and white arrowhead shows the nascent spike branch. The blue arrowhead in (f) shows additional spikelet meristem in place of central glume. (k–o) *vrs3vrs5*: (k) basal node bearing a cluster of awned spikelets; (l) triplet from middle node of spike base; (m) SEM of late SP stage; (n) base of AP spike and (o) WA spike. Orange shading in (j) marks early multi‐floreted spikelet. Yellow shading (f, j, m–o) shows basal additional band and subsequent spikelet meristems. (p–r) Mean normalised expression (mne) determined by qPCR on spikes from glume primordium (GP), LP and SP‐staged spikes for: (p) *Barley Meristem 3* (*BM3*) and *Barley Meristem 8* (*BM8*), ± SE; (q) *HvLONELY GUY1* (*HvLOG1*) and *HvCYTOKININ OXIDASE2* (*HvCKX2*), ± SD; and (r) *VRS5* and *VRS1*, ± SD; (*n* = 3). Significant differences to Bowman indicated by *, *P *<* *0.05; **, *P *<* *0.01; ***, *P *<* *0.001 (Student's *t*‐test). Scales (a–c, k, l), 0.5 cm; (d–j, m–o), 100 μm.

In addition to gains in lateral spikelet fertility, novel *vrs* combinations also increased additional spikelet production. While *vrs1vrs3*,* vrs1vrs5* and *vrs4vrs5* spikes showed modest increases in additional spikelet formation from between one to four (Table S1), *vrs3vrs4* spikes generated 19 (± 9.1) additional spikelets and one to two ectopic spike branches from middle nodes, similar to *vrs4.k* (Figs 4a,b, 5a–c; Table S1). These phenotypes may reflect a delayed or weakened progression from TSM to determinate spikelet and floret in *vrs3vrs4* spikes, manifested by altered meristem identity and determinacy. First, the CSMs at GP‐LP transition elongated to form an abaxial boundary ridge and subsequent axillary meristem which could adopt branch identity (Fig. 5d–g). Second, axillary meristems formed within the glume axil of central and lateral spikelets (Fig. 5d,f), sometimes consuming the glume into a spikelet (Fig. 5c). Third, secondary spikelet meristems developed on the basal border of the lateral spikelets’ outer glume which could also consume the glume into an ectopic spikelet (Fig. 5f,g,i). Fourth, floret organ differentiation was noticeably delayed in LP to SP spikelet meristems compared with Bowman (Fig. 5f–h). Last, both canonical and additional spikelets could generate more than one floret and extra floral organs (Fig. 5j). The *vrs3vrs4* double mutant also formed a continuous indeterminate lateral meristematic ridge between glume primordia of the central spikelet on the basal node which then split into additional spikelet meristems (Fig. 5f,i,j), fuelling the large increase in spikelet number at the basal node (Fig. 4; Table S2), a phenotype not observed in *vrs4.k*. Basal nodes of *vrs3vrs5* spikes also developed clusters of additional spikelets and mutlifloreted spikelet bouquets with extra glumes (Figs 4a,b, 5h,i), similarly developing from an enlarged indeterminate basal meristem (Fig. 5m–o). Thus, both *vrs3vrs4* and *vrs3vrs5* spikes displayed meristem identity and determinacy defects.

To explore this further, we examined the expression of genes involved in determining meristem fate. In agreement with the *vrs3vrs4* and *vrs3vrs5* phenotypes, we found that transcripts of *APETALA1/FRUITFULL* homologue spikelet/FM identity genes, *BM3* (*HvMADS18*) and *BM8* (*HvMADS15*) (Schmitz *et al*., 2000; Trevaskis *et al*., 2007) were almost completely undetectable in *vrs3vrs5* at LP (*P* < 0.05), while *BM3* was 67% reduced in *vrs3vrs4* (*P* < 0.05; 61% decline in BM8 was *P* > 0.05 for *vrs3vrs4*; Fig. 5p; Table S5). Furthermore, elevated transcripts of *HvLONELY GUY1*, which encodes a cytokinin biosynthetic enzyme, in *vrs3vrs4* (*P* < 0.05) and at later stages for *vrs3vrs5* (*P* < 0.005) which, coupled with lower *HvCYTOKININ OXIDASE* levels at most stages examined (*P* < 0.05), suggested a misregulation of cytokinin metabolism (Fig. 5q; Table S5), previously associated with additional spikelet and FMs in rice and barley (Han *et al*., 2014; Youssef *et al*., 2017). Expression of the barley orthologue of *RAMOSA ENHANCER LIKE2* (*REL2*) was 50% reduced at LP (*P* < 0.05) in both double mutants (Fig. S4; Table S5), consistent with enhanced indeterminacy of maize *rel2* mutants (Gallavotti *et al*., 2010). Notably, *HvINDETERMINATE SPIKELET1* (*HvIDS1*) expression doubled at LP in *vrs3vrs4* (*P* < 0.05; Fig. S4; Table S5). Ectopic expression of *ZmIDS1* in maize is associated with extra FMs (Chuck *et al*., 2008). Taken together, these changes in gene expression are consistent with altered meristem fate in *vrs3vrs4* and *vrs3vrs5*.

As many features of *vrs3vrs4* resembled *vrs4.k* (Fig. 1), we also examined whether loss of *VRS4* expression in *vrs3vrs4* may explain its severe phenotypes. While reduced in *vrs3vrs5* at GP, *VRS4* expression was unaltered in *vrs3vrs4* (Fig. S4; Table S5). This may reflect the increase in meristematic tissue load on these spikes, such that *VRS4* level per meristem is actually reduced, or that these phenotypes are independent of effects on *VRS4* expression. Both *vrs3vrs4* and *vrs3vrs5* had basal spikelet clusters, so we also hypothesised that downregulation of *VRS5* in *vrs3vrs4* may contribute to this phenotype. *VRS5* in *vrs3vrs4* was reduced by 91% at SP (*P* < 0.005) and 97% at AP (*P* < 0.005; Fig. 5r), far greater than either parent (Table S5), agreeing with a role for both VRS3 and VRS4 in *VRS5* induction. Reduced *VRS2* expression at LP in *vrs3vrs5* (50%, *P* < 0.005) is also consistent with extra spikelet production in basal nodes (Fig. S4; Table S1). *VRS1* expression was extremely low in both double mutants, in agreement with their awned, fertile spikelets (Fig. 5r, *P* < 0.0005).

### Combined variation in *VRS3*,* VRS4* and *VRS5* associated with improved grain parameters

**Figure 6:**
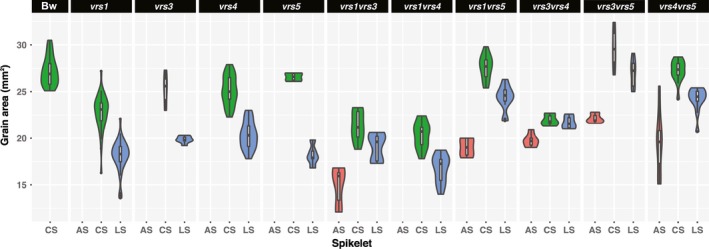
Grain area for grain from lateral spikelets, central spikelets and additional spikelets from *Hordeum vulgare* ssp. *vulgare* Bowman (Bw), single *vrs* mutants and double *vrs* mutants. Violin plots show grain area distribution. Central spikelets shown in green, lateral spikelets shown in blue and additional spikelets shown in orange. CS, central spikelet; LS, lateral spikelet; AS, additional spikelet.

We next assessed whether novel *vrs* allelic combinations improve either grain weight and/or homogeneity compared with parents. We first measured grain parameters across the single mutant lines which showed decreased average grain area and reduced TGW compared to Bowman, highlighting a well known trade‐off between grain size and number (Liller *et al*., 2015; Table 2). However, *vrs3*,* vrs3.f*,* vrs4* and *vrs5* alleles had lower grain number per spike compared with *vrs1* yet similar TGW (Tables 2, S4, S6), due to decreased lateral grain weight (Fig. 6; Table S7), suggesting that the relationship between TGW and grain number/spike is influenced by size differences between central and lateral grain, which varied amongst row‐type alleles. For instance, *vrs1vrs3* and *vrs1vrs4* set more or equal grain per spike as *vrs1*, and showed a lower or equivalent TGW compared with parents (Tables 2, S8). By contrast, despite increases in grain number per spike, *vrs5* in combination with *vrs1*,* vrs3* or *vrs4*, either increased grain area and/or TGW (Table S4) due to increased area, length, width and TGW of lateral grain to be equivalent to central grain of the single *vrs5* parent (Fig. 6; Table S8). Central grain area and width of *vrs3vrs5* double mutants exceeded the *vrs5* parent (Fig. 6; Table S8), and most strikingly, significantly increased compared with Bowman (Fig. 6; Table S8). Additional spikelet grain from *vrs3vrs5* was smaller compared with central and lateral grain (Table S8), but they contributed little to total grain (3.4%), such that overall grain area and TGW in *vrs3vrs5* were equivalent to Bowman (Table 2). By contrast, pairing *vrs3* with *vrs4* decreased the area, length and weight of central grain to equal that of lateral and additional grain (Fig. 6; Table S8), potentially due to the very high spikelet load per spike. As grain from additional spikelets of *vrs3vrs4*, which contributed 18% of total grain (Table S1), showed equivalent weight to other spikelet grain resources may be more evenly distributed across *vrs3vrs4* spikelets than other *vrs* mutants with additional spikelets which showed lower weight compared to central and lateral grain (Table S8). Decreased central grain weight in *vrs3vrs4* reduced the central: lateral area ratio to 1.01 (± 0.1) while increased additional spikelet grain weight reduced the central: additional spikelet grain ratio to 1.11 (± 0.04) (Tables 3, S8). Taken together, entire grain lots from *vrs3vrs4* showed superior grain homogeneity compared with all other lines (Fig. S5).

**Table 3:** Grain area uniformity in double *vrs* mutants and their parents

Line	Central : lateral	Central : additional
*vrs1*	1.26 ± 0.12	na
*vrs3*	1.27 ± 0.09	na
*vrs4*	1.23 ± 0.10	na
*vrs5*	1.45 ± 0.08	na
*vrs1vrs3*	1.12 ± 0.04***(*vrs1*)/***(*vrs3*)	1.49 ± 0.32
*vrs1vrs4*	1.22 ± 0.05	na
*vrs1vrs5*	1.14 ± 0.04**(*vrs1*)/***(*vrs5*)	1.45 ± 0.17
*vrs3vrs4*	1.01 ± 0.07***(*vrs3*)/***(*vrs4*)	1.11 ± 0.04
*vrs3vrs5*	1.10 ± 0.07**(*vrs3*)/***(*vrs5*)	1.34 ± 0.09
*vrs4vrs5*	1.13 ± 0.05**(*vrs4*)/***(*vrs5*)	1.41 ± 0.25

Grain area uniformity calculated as a ratio of central grain area to lateral or additional grain area in *Hordeum vulgare* ssp. *vulgare* double *vrs* mutants and their parents. Values are mean (± SD) from grain harvested from *n* = 10 spikes per line. Significant differences between double *vrs* mutants and single *vrs* mutant parents by **, *P *<* *0.01; ***, *P *<* *0.001 (Student's *t*‐test). na, not applicable.

**Table 4:** Whole plant traits and phenology for Bowman (Bw), single *vrs* mutants and double *vrs* mutants

Line	Final tiller number	Heading (dag)	Plant height (cm)	Spike length (cm)
Bw	27.0 ± 3.2	38.6 ± 1.8	92.3 ± 9.5	8.90 ± 0.58
*vrs1*	21.3 ± 2.3**	**39.2 ± 3.2**	**91.3 ± 4.9**	8.60 ± 0.45
*vrs2*	36.8 ± 2.8***	45.4 ± 3.2	**88.7 ± 7.6**	9.20 ± 0.6
*vrs3.f*	22.5 ± 2.6**	43.4 ± 2.9	**90.1 ± 4.1**	9.00 ± 0.43
*vrs3*	21.6 ± 2.1***	41.5 ± 2.2	**85.4 ± 10**	8.37 ± 0.64
*vrs4.k*	18.0 ± 1.4***	**39.0 ± 3.2**	100 ± 2.0*	7.80 ± 0.48***
*vrs4*	19.3 ± 5.2***	48.3 ± 6.0***	105 ± 8.8**	8.45 ± 0.55
*vrs5*	17.6 ± 2.9***	44.7 ± 3.8***	**86.2 ± 7.0**	8.03 ± 0.53***
*vrs1vrs3*	21 ± 3.7**	45.6 ± 4.6***	**101 ± 14**	9.22 ± 0.87
*vrs1vrs4*	22.2 ± 5.5**	**39.9 ± 1.8**	**90.2 ± 6.9**	7.95 ± 0.66
*vrs1vrs5*	11.5 ± 2.8***	42.8 ± 1.3***	84.3 ± 4.9*	7.64 ± 0.26***
*vrs3vrs4*	**26.1 ± 6.1**	51.9 ± 5.2***	**86.1 ± 7.0**	9.66 ± 0.79*
*vrs3vrs5*	8.95 ± 2.0***	57.6 ± 11***	82.8 ± 7.0**	8.12 ± 0.61
*vrs4vrs5*	13.2 ± 2.0***	44.8 ± 5.7***	101 ± 8.3*	7.98 ± 0.55***

Whole plant traits and phenology in *Hordeum vulgare* ssp. *vulgare* single *vrs* mutants, double *vrs* mutants and Bowman. Values are the mean (± SD) from *n* = 10 individuals per line. Bold text indicates no significant difference with Bowman. Significant differences with Bowman indicated by *, *P *<* *0.05; **, *P *<* *0.01; ***, *P *<* *0.001 (Student's *t*‐test). dag, days after germination.

### Row‐type allelic variation shows differential effects on tillering

In addition to reducing TGW, we noted that row‐type alleles also increased the proportion of fully‐awned, empty spikelets compared with Bowman (Table 2), consistent with a reduction in spikelet survival observed for six‐rowed varieties (Alqudah & Schnurbusch, 2014). The proportion of empty awned spikelets was particularly high in *vrs3vrs4* spikes, potentially due to resources diverted to ectopic branches and many additional spikelets, as well as additional rachis nodes (Table 2). However, other architectural changes influenced by row‐type alleles such as the outgrowth of axillary basal buds or tillers, could be acting as competing energy sinks (Elalaoui *et al*., 1988; Gu & Marshall, 1988).

**Figure 7:**
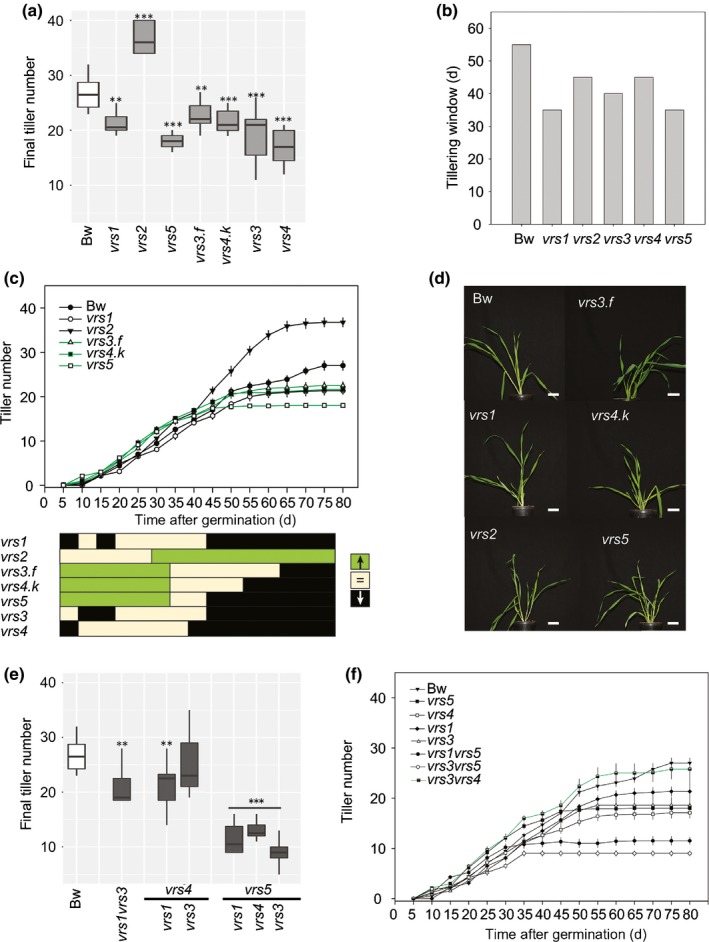
Tiller outgrowth across *Hordeum vulgare* ssp. *vulgare* Bowman (Bw), single *vrs* mutants and double *vrs* mutants. (a) Final tiller number at harvest for Bowman and single *vrs* mutants. Horizontal bars in each box represent mean values (± SE) calculated from main culm spikes (*n* = 7–12). (b) Tillering window in single *vrs* mutants. (c) Tiller outgrowth during development in Bowman and single *vrs* mutants and selected double mutants. Dots represent mean values (± SE) calculated from main culm spikes (*n* = 7–12). Significant differences between Bowman and *vrs* mutants indicated by lower colour panel (*P* < 0.05). Green indicates statistical increase in tiller number, beige shows no difference and black indicates a statistical decrease in tiller number, compared with Bowman. (d) Pictures of single *vrs* mutants 28 d after sowing. (e) Final tiller number determined at harvest for Bowman and double *vrs* mutants. Horizontal bars in each box represent mean values (± SE) calculated from main culm spikes (*n* = 7–12). (f) Tiller outgrowth during development single *vrs* mutant parents and selected double mutants. Significant differences to Bowman in (a) and (e) indicated by *, *P *<* *0.05; **, *P *<* *0.01; ***, *P *<* *0.001 (Student's *t*‐test). Box plot horizontal line denotes the mean, while the box shows the 95% confidence interval. Bars, (d) 1 cm. Bw, Bowman.

To explore, we first assessed tillering in single *vrs* mutants compared Bowman. Other than *vrs2*, all single mutants generated fewer tillers at maturity and tillered for a shorter period of time (Figs 7a,b, S6; Table 4). The tillering window was especially curtailed in *vrs4.k* and *vrs5* which ceased tillering 20 d earlier than Bowman at 45 dag. Although tillers emerged at either that same (*vrs1*,* vrs3* and *vrs5*, 0.47 tillers per day) or reduced rate (*vrs4*, 0.4 tillers per day) as Bowman, the *vrs3.f* mutant developed 0.5 tillers per day and the rate was even higher in *vrs2* and *vrs4.k* at 0.72 and 0.54 tillers per day, respectively (Fig. S6). We analysed this trait further by comparing tiller number in *vrs* mutants to Bowman at 5 d intervals following germination (Fig. 7c). The *vrs3.f*,* vrs4.k* and *vrs5* mutants had more tillers until 40 dag, after which tiller number was equivalent, only becoming lower than Bowman as tillering stopped in these lines and continued in Bowman. By contrast, *vrs2* showed a higher tiller number only at and after 35 dag, suggesting a later tillering effect. Conversely, *vrs1*,* vrs3* and *vrs4* did not lead to increased tiller number at any stage but reduced tiller number at or shortly after the end of their tillering window. Early cessation of tillering was not associated with earlier heading times, which were either equivalent or later than Bowman, or with changes in plant height (Table 4). In summary, row‐type alleles fell into one of three classes of tiller outgrowth compared with the two‐rowed parent: (1) promotion of early tillering followed by early cessation (*vrs3.f*,* vrs4.k* and *vrs5*); (2) early cessation only (*vrs1*,* vrs3*,* vrs4*); or (3) promotion of late tillering (*vrs2*). Repeating this experiment in smaller pots with more resolution at earlier time points along with apex inspection, we found that tillers emerged earliest in *vrs4.k* and *vrs5*, followed by *vrs3.f*, when the apex was still a vegetative dome (Fig. S7). So, elevated tiller number at early stages may reflect an earlier release of bud dormancy compared with other lines. Notably, row‐type alleles linked to early increases in tillering rate (*vrs4.k*,* vrs3.f*, and *vrs5*) showed a longer TSM duration and were also associated with additional spikelet production (Fig. S7).

We next examined whether combining *vrs* alleles within and between classes altered tiller number (Fig. 7f; Tables 4, S4). The *vrs1vrs3* and *vrs1vrs4* mutants generated fewer tillers at harvest compared with Bowman, equivalent to their parental lines. Double mutants with *vrs5* showed reduced tiller number compared with both parents. Suppression in tiller number may compensate for increased fertile spikelet load per spike and may explain why *vrs1vrs5* and *vrs4vrs5* double mutants showed an equivalent number of empty awned spikelets as Bowman (Table 2). However, *vrs3vrs5* plants were especially deficient in tillers (Fig. 7e,f; Table S4), reflecting both the shortest tillering window (25 d) and lowest overall rate (0.31 tillers per day; Figs 7f, S6) across all genotypes, despite having the latest heading date, suggesting that resources diverted to tillering may not be linked to the substantial loss of grain set in otherwise fertile *vrs3vrs5* spikelets. In striking contrast with *vrs3vrs5*,* vrs3vrs4* plants had the same tiller number as Bowman, increased compared with their parents and *vrs4.k* (Fig. 7f; Tables 2, S4). As the tillering window of *vrs3vrs4* was unchanged compared with their parents, the increased tiller number reflected a higher overall tillering rate (0.53 tillers per d; Figs 7f, S6). Increased tillering coincides with stem elongation to heading (Table 4), when spikelets are particularly susceptible to abortion (Alqudah *et al*., 2016), which may contribute to absence of grain in otherwise fertile spikelets, in addition to the ectopic awned spikelet and floret production in *vrs3vrs4*.

## Discussion

### Developmental basis for awn association with lateral fertility

Our work suggests that *VRS1* acts as a downstream integrator of VRS3, VRS4 and VRS5 to repress both lateral spikelet fertility and awn development. Coordinated initiation of carpel and awn primordia in larger lateral spikelets may reflect a linked step in the spikelet developmental programme, the ‘vital connection’ speculated by Brenchley (1920), specifically targeted by *VRS1*. Carpels initiate when the central FM differentiates into a determinate organ primordium (Smyth *et al*., 1990; Schoof *et al*., 2000) and the AP itself may represent a proliferating ‘quasi meristem’ (Girin *et al*., 2009; Toriba & Hirano, 2014). Therefore, *VRS1* may block carpel and awn initiation at AP stage by inhibiting localised meristem proliferation, leading to a dormant‐like state. Indeed, *VRS1* was recently shown to repress proliferation in both leaf and lateral spikelet primordia (Sakuma *et al*., 2017; Thirulogachandar *et al*., 2017). Lateral spikelets in a different *vrs1* mutant to that studied here, *vrs1.c*, are fully fertile, yet only develop very short lemma extensions (Konishi & Franckowiak, 1997; Ullrich, 2011), suggesting *VRS1* control of lateral spikelet fertility and awn development can be uncoupled. As *vrs1.c* contains no coding sequence mutations (Saisho *et al*., 2009), potential differential *cis*‐regulation between the awn and carpel may be relevant.

### Lateral spikelet in/fertility shows parallels with sex determination in maize

Lateral spikelets in two‐row barley have rudimentary stamens yet exceptionally reduced ovaries and are sometimes referred to as staminate (Beavan, 1902; Brenchley, 1920; Komatsuda *et al*., 2007), a state that may originate at the AP stage when infertile lateral spikelets initiate stamen but not carpel primordia (Fig. 2). Stamen growth appeared particularly robust in *vrs5* mutants and our previous work showed that grain set in *vrs5* (*Intermedium‐c)* mutants in a functional *VRS1* (*Vrs1.b*) background was correlated with lateral spikelet male fertility (Ramsay *et al*., 2011). We surmise that *VRS5* may be more important to repress male fertility, while initiation of female organs may be more sensitive to *VRS1*. The possibility of sex‐specialised regulation by *VRS1* and *VRS5* shows an overlap with their homologues in sex determination of the maize ear and tassel. The maize *VRS1* homologue, *grassy tillers1* (*gt1*), promotes carpel abortion to generate the distinctive staminate spikelets of the tassel, while loss of function leads to carpel development and feminised tassels (Whipple *et al*., 2011). Conversely, reduced expression of the maize *VRS5* orthologue *teosinte branched 1* (*tb1*) causes tassel‐tipped ears and conversion of ear spikelets to tassel spikelets, suggesting that *tb1* excludes male identity from the ear (Doebley *et al*., 1995; Hubbard *et al*., 2002). Differential expression probably contributes to sex determination, with *tb1* expression strongly enhanced in stamen primordia of the ear spikelet and *gt1* specifically expressed in carpels of the tassel spikelet (Hubbard *et al*., 2002; Whipple *et al*., 2011). By contrast, *VRS1* is expressed in all lateral spikelet organs, although enriched in developing carpels and lemmas (Sakuma *et al*., 2013). The *VRS5* expression pattern is yet unknown and it would be interesting to determine whether the role of *VRS5* in male infertility is associated with increased expression in stamen vs carpel primordia.

### VRS3 as an epigenetic modifier of row‐type gene function

Loss of *VRS3* function along with defective *VRS4* or *VRS5* alleles caused synergistic losses in meristem determinacy, potentially by regulating their expression as well as that of shared regulatory targets. Lundqvist & Lundqvist (1988) described the modifier ability of *vrs3* as having a ‘rigid’ nature that forced other alleles to ‘show their hands’. Consistent with this type of control, *VRS3* encodes a putative Jumonji C‐type H3K9me2/me3 demethylase (Bull *et al*., 2017; van Esse *et al*., 2017), predicted to control gene expression through regulating chromatin state. An epigenetic mode of action is also consistent with the phenotypic instability conferred by *vrs3* alleles (Bull *et al*., 2017) and reported for lateral spikelets and awns in general (Engledow, 1921). The *vrs3vrs4* and *vrs3vrs5* double mutants showed fully six‐rowed phenotypes associated with a complete loss of *VRS1* expression. *VRS5* contributes modestly to *VRS1* induction, so VRS3 probably mediates an additional inductive effect on *VRS1*. While this mechanism remains unknown, *VRS3* could act epigenetically to stabilise local *VRS1* chromatin in an active transcriptional state, similar to the spatial regulation of Drosophila *Hox* genes (Bantignies & Cavalli, 2006; Ringrose & Paro, 2007), that may buffer against impaired *VRS4* and *VRS5* function. Furthermore, although *VRS4* is key to *VRS1* induction (Koppolu *et al*., 2013; this paper), *VRS4* transcripts significantly decline, especially at AP and later stages while *VRS1* levels increase (Koppolu *et al*., 2013; Sakuma *et al*., 2013; Fig. 3), suggesting that another factor, which could involve *VRS3*, maintains *VRS1* expression. In maize, the paramutator Rmr6 locus is required to maintain epigenetic states conferred by long noncoding RNA molecules to prevent carpel emergence in tassels (Parkinson *et al*., 2007). It will be important to define the chromatin modifications associated with *VRS3* activity and whether DNA methylation and long noncoding RNAs are involved in *VRS3*‐mediated epigenetic regulation.

### Lateral spikelet fertility regulators influence meristem identity and determinacy

Alteration in the timing of meristem transitions and meristem maturation is associated with variation in inflorescence branching and floral architecture across plants (McKim & Hay, 2010; Yoshida *et al*., 2013; Park *et al*., 2014; Lemmon *et al*., 2016; Ta *et al*., 2017). The barley inflorescence forms as the lateral meristems transition from DR to TSM to SM to FM identity. In *vrs3vrs4*, indeterminate inflorescence branches bud from the CSM, potentially due to disrupted progression of this pathway. Similarly, multiple FMs within the *vrs3vrs4* and *vrs3vrs5* spikelets may reflect a delayed transition from SM (capable of giving rise to florets) to FM, while extra floral organs suggest a problem in determinate floret identity. Branches do not form at the DR or TSM stage of *vrs3vrs4* or *vrs4.k*, so pathways promoting DR and TSM identity may be less sensitive to defective *VRS4* or *VRS3* function. Rather, these genes may be more important for promoting the transition to determinate spikelet meristem identity and/or repress branching identity, especially in the central spikelet. Branches also do not develop from LSMs, suggesting that spikelet identity may be more complete by the time of LSM transition, which occurs after CSM determination. However, *vrs3vrs4* lateral spikelets initiate extensive flanking secondary spikelets, often off, or at the expense of, the glume (an event also observed in central spikelets), which could represent a shift to indeterminacy after spikelet identity is established (Bommert & Whipple, 2018). In *vrs3vrs5* and *vrs3vrs4*, basal indeterminate lateral meristems develop between central spikelet glumes, leading to additional spikelets with extra floral organs later in spike development, consistent with delayed acquisition of determinate spikelet and floret fate and aberrant glume development. We detected the lower expression of spikelet meristem identity genes *BM3* and *BM8* in *vrs3vrs4* and *vrs3vrs5* mutants and we suspect that other genes known to regulate spikelet identity and/or determinacy in barley or other grasses, such as *COMPOSITUM2*, could also be misregulated, as shown for *vrs4.k* (Poursarebani *et al*., 2015). Additional spikelet development observed in basal nodes of *vrs2* spikes was associated with disrupted hormone gradients characterised by increased cytokinin and lower auxin levels (Youssef *et al*., 2017). VRS3 and VRS5 may also be important to establish hormone gradients to properly pattern the spike, as supported by reduced *VRS2* expression and misexpression of cytokinin metabolic genes in *vrs3vrs5*.

While *VRS3*,* VRS4* and *VRS5* may work through *VRS1* to repress lateral spikelet growth, *vrs1vrs5*,* vrs1vrs3* and *vrs1vrs4* double mutants showed only a small increase in additional spikelet formation that, along with *VRS1* temporal and spatial expression patterns, suggested a minor role for *VRS1* in spikelet meristem identity and determinacy. The paralogue of *VRS1*,* HvHOX2* is strongly expressed in the barley rachis vasculature (Sakuma *et al*., 2010, 2013), as is *VRS3* (Bull *et al*., 2017), while *VRS4* is localised in the rachis and the spikelet−rachis boundary (Koppolu *et al*., 2013). Increased expression of *gt1* (*VRS1* orthologue) in the vascular nodal plexus in maize prevents secondary ear formation (Wills *et al*., 2013). Given these partially overlapping expression patterns, *VRS3*,* VRS4* and/or *VRS5* gene function may promote expression of *HvHOX2* in rachis node either directly on nonautonomously from the spikelet boundary to promote determinacy and identity of the adjacent meristem, which may involve signalling from the boundary, as suggested for the *ramosa* pathway in maize, and/or from the suppressed bract as suggested by the *plastochron* mutants in rice (Bommert & Whipple, 2018).

Other mutants with spike branching and extra spikelets, such as *compositum1* and *poly‐and‐branched‐row spike* also increase lateral spikelet development (Larsson, 1985; Shang *et al*., 2014) that, taken with the results here, suggest a profound link between control of LSM growth and spikelet and floret identity. Dixon *et al*. (2018) showed that increased dosage of *TB1* causes a paired spikelet phenotype in wheat (where an extra spikelet forms immediately beneath the primary spikelet) associated with reduced spikelet meristem identity gene expression and delayed spikelet meristem maturation. Dixon *et al*. (2018) speculated that VRS5 (HvTB1) similarly blocks lateral spikelet progression to floral fate, thereby leading to bud dormancy (and lateral spikelet infertility) and predicted that impaired *vrs5* alleles release lateral spikelet dormancy by enabling expression of floral genes. Our ontogenetic analysis suggests that lateral spikelets in two‐rowed spikes are blocked at carpel emergence, consistent with an arrest of the floral programme, but also that these LSMs are smaller at the onset of floral differentiation. Acquisition of determinate floral fate must be carefully timed to allow floral meristems to proliferate sufficiently to fuel a full complement of floral organs (McKim & Hay, 2010). Our data suggest that *vrs5*, as well as *vrs4.k* and *vrs3.f*, alleles prolong the TSM phase, potentially allowing increased proliferation before acquisition of spikelet fate. A comparison of the effects of *TB1* in wheat and *VRS5* in barley on stage‐specific meristem maturation transcriptomes may help to understand the divergence and conservation of the pathways that regulate inflorescence to spikelet to floret identity in the Triticeae.

### Tillering and VRS5

In maize, TB1 induction of *gt1* is necessary to promote apical dominance, especially under unfavourable conditions (Whipple *et al*., 2011). In barley, however, *VRS5* appears less important for *VRS1* expression, and defects in both genes are associated with fewer tillers, due to a shortened tillering window compared with the characteristic ‘bushy’ phenotype of *tb1* and *gt1* mutants (Doebley *et al*., 1995; Whipple *et al*., 2011). It will be interesting in the future to examine whether axils without visible tillers have underdeveloped, multiple or dormant tiller buds. Previous studies in barley showed an early increase in tillering in *vrs5* (Ramsay *et al*., 2011; Liller *et al*., 2015) that we also detected here, suggesting that weakened *VRS5* function is associated with an early release of tiller bud dormancy. Unlike *vrs1* and *vrs5*, mutations in *gt1* and *tb1* are not reported to increase grain number in the inflorescence.

### Novel routes to grain improvement

Grain yield and quality is the cumulative output of the barley life cycle and is reflective of source/sink relationships in the context of developmental time (Slafer, 2003). Altering the balance of these latter relationships could therefore impact components of yield. Indeed, our data show that different single and double *vrs* alleles introduce perturbations in the numbers of fertile spikelets per spike, spikelet uniformity, grain size and/or the number of spikes per plant through increased, decreased or arrested tillering. As an example, the early tillering arrest we observed in *vrs5* plants could reflect resource allocation to spikes with an increased number of fertile spikelets. However, we believe this is unlikely as multiple lines, such as *vrs1.a* and *vrs4.k*,* vrs1vrs3*,* vrs3vrs4*, had both more grain‐setting spikelets per spike and tillers compared with *vrs5*, suggesting that changes in tillering due to defective *vrs* gene function is unlikely to be a simple read out of energy demand. Of relevance here is the *deficiens* allele of *VRS1 (Vrs1.t1*, Sakuma *et al*., 2017) that has recently had a significant effect on barley breeding in NW Europe. *Vrs1.t1* almost completely curtails development of lateral spikelets and is associated with increased cell proliferation in the central spikelets that may promote larger grains and higher productive yield potential by allocating more resources directly to the central grain (Sakuma *et al*., 2017). In *vrs3vrs4* and *vrs3vrs5* double mutants (containing wild‐type *VRS1*) we observed improved grain homogeneity, an important quality characteristic of malting barley. From a developmental perspective, this may reflect early equivalency in floral organ size that may have practical value in end‐use quality improvement. Our investigations therefore revealed both shared and independent roles for *VRS* genes in conferring lateral spikelet infertility, meristem determinacy and divergence in tillering regulation, and may provide novel routes to improving six‐row barley grain quality. Our recent work showing improved grain parameters in *vrs1.a Int‐c.a vrs3*, as described in Bull *et al*. (2017), highlights efforts to integrate novel row‐type alleles into the cultivated germplasm.

## Author contributions

SMM and RW conceived the research. SMM, RW and MZ designed the research. MZ performed the research and analysed the data. SMM, RW and MZ wrote the manuscript.

## Supporting information

Please note: Wiley Blackwell are not responsible for the content or functionality of any Supporting Information supplied by the authors. Any queries (other than missing material) should be directed to the *New Phytologist* Central Office.

**Fig. S1** Spikes of Bowman and single *vrs* mutants before awn removal.
**Fig. S2** Spikes of *vrs3.f*,* vrs4*,* vrs5*,* vrs1* and Bowman at late awn primordium stage.
**Fig. S3** Spikes of *vrs* double mutants before awn removal.
**Fig. S4** Differential gene expression of meristem regulators in Bowman, *vrs3vrs4* and *vrs3vrs5* mutants.
**Fig. S5** Combined grain area from Bowman, single *vrs* mutants and double *vrs* mutants.
**Fig. S6** Tiller outgrowth rate over time in Bowman and single *vrs* mutants and double *vrs* mutants.
**Fig. S7** Early tillering phase across Bowman and single *vrs* mutants.
**Method S1** Supplemental methods for genotyping and qRT‐PCR.

**Table S1** Spike and spikelet traits separated by central, lateral and additional spikelets in Bowman, single *vrs* mutants and double *vrs* mutants.
**Table S2** Spikelet by rachis node for Bowman, single *vrs* mutants and double *vrs* mutants.
**Table S3** *VRS* gene expression in Bowman and single *vrs* mutants.
**Table S4** Comparison of spikelet traits between double *vrs* mutants, their parents and Bowman.
**Table S5** Gene expression in Bowman, *vrs3vrs4* and *vrs3vrs5* double mutants and *vrs3*,* vrs4* and *vrs5* parents.
**Table S6** Spikelet parameters in single *vrs* mutants and Bowman compared by ANOVA.
**Table S7** Grain parameters in *vrs* single mutants and Bowman compared by ANOVA.
**Table S8** Grain parameters in *vrs* double mutants compared with their parents and Bowman by ANOVA.
