# Supplementary material for: Interaction between row‐type genes in barley controls meristem determinacy and reveals novel routes to improved grain
Source: New Phytol. 2018 Nov 27;221(4):1950–65. doi: 10.1111/nph.15548 (PMC6492131; doi:10.1111/nph.15548)
Supplement: Supplementary file 1 — Fig. S1 Spikes of Bowman and single vrs mutants before awn removal. Fig. S2 Spikes of vrs3.f, vrs4, vrs5, vrs1 and Bowman at late awn primordium stage. Fig. S3 Spikes of vrs double mutants before awn removal. Fig. S4 Differential gene expression of meristem regulators in Bowman, vrs3vrs4 and vrs3vrs5 mutants. Fig. S5 Combined grain area from Bowman, single vrs mutants and double vrs mutants. Fig. S6 Tiller outgrowth rate over time in Bowman and single vrs mutants and double vrs mutants. Fig. S7 Early tillering phase across Bowman and single vrs mutants. Method S1 Supplemental methods for genotyping and qRT‐PCR. [file NPH-221-1950-s001.pdf]

New Phytologist **Supporting Information**
Article title: Interaction between row-type genes in barley controls meristem determinacy and reveals novel routes to improved grain
Authors: Monika Zwirek, Robbie Waugh and Sarah M. McKim
Article acceptance date: 22 September 2018

The following Supporting Information is available for this article:

Fig. S1 Spikes of Bowman and single *vrs* mutants before awn removal
Fig. S2 Spikes of *vrs3.f*, *vrs4*, *vrs5*, *vrs1* and Bowman at late awn primordium stage
Fig. S3 Spikes of *vrs* double mutants before awn removal
Fig. S4 Differential gene expression of meristem regulators in Bowman, *vrs3vrs4* and *vrs3vrs5* mutants.
Fig. S5 Combined grain area from Bowman, single *vrs* mutants and double *vrs* mutants.
Fig. S6 Tiller outgrowth rate over time in Bowman and single *vrs* mutants and double *vrs* mutants
Fig. S7 Early tillering phase across Bowman and single *vrs* mutants

Table S1 Spike and spikelet traits separated by central, lateral and additional spikelets in Bowman, single *vrs* mutants and double *vrs* mutants (separate excel file)
Table S2 Spikelet by rachis node for Bowman, single *vrs* mutants and double *vrs* mutants (separate excel file)
Table S3 *VRS* gene expression in Bowman and single *vrs* mutants (separate excel file)
Table S4 Comparison of spikelet traits between double *vrs* mutants, their parents and Bowman (separate excel file)
Table S5 Gene expression in Bowman, *vrs3vrs4* and *vrs3vrs5* double mutants and *vrs3*, *vrs4* and *vrs5* parents (separate excel file)
Table S6 Spikelet parameters in single *vrs* mutants and Bowman compared by ANOVA
Table S7 Grain parameters in single *vrs* mutants and Bowman compared by ANOVA (separate excel file)
Table S8 Grain parameters in double *vrs* mutants compared to their parents and Bowman by ANOVA (separate excel file)
Methods S1 Supplemental methods for genotyping and qPCR

Fig. S1 Spikes of Bowman and single *vrs* mutants before awn removal. Scale, 0.5 cm.

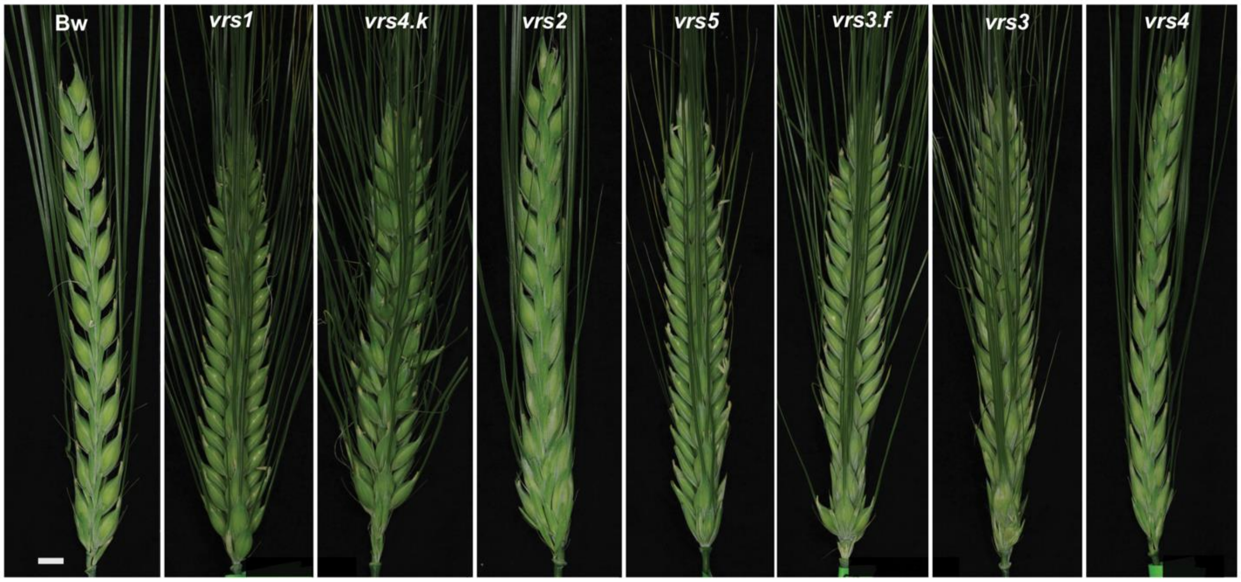

Fig. S2 Spikes of *vrs3.f*, *vrs4*, *vrs5*, *vrs1* and Bowman at late awn primordium stage. Scanning electron microscopy of spikes at late awn primordium (AP) stage from *intermedium* alleles: **(a)** *vrs3.f*, **(b)** *vrs4*, **(c,d)** *vrs5*, **(e)** *vrs1*, **(f)** Bowman. Lemma and awns are false coloured to highlight the gradient in development along the intermdium spikes. Arrow in (d) shows variable lemma and awn development in *vrs5*. Arrowheads in (d-f) show differentiated stamen lobes in *vrs5* lateral spikelets compared to *vrs1* and Bowman. Scale, 100 µm. Bw, Bowman

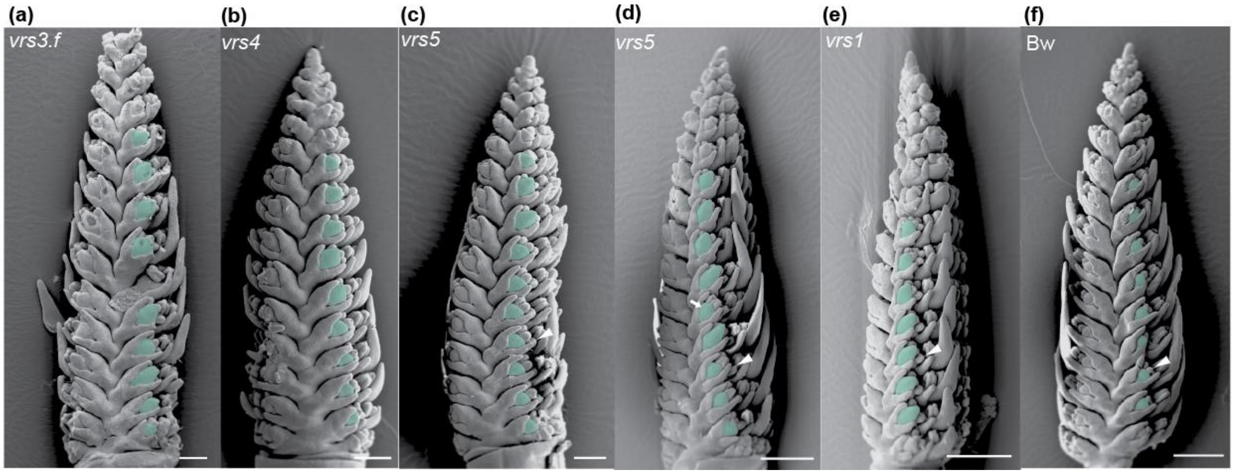

Fig. S3 Spikes of *vrs* double mutants before awn removal. Genotypes from left to right: *vrs1vrs3*, *vrs1vrs4*, *vrs1vrs5*, *vrs3vrs4*, *vrs3vrs5*, and *vrs4vrs5.* Scale, 0.5 cm.

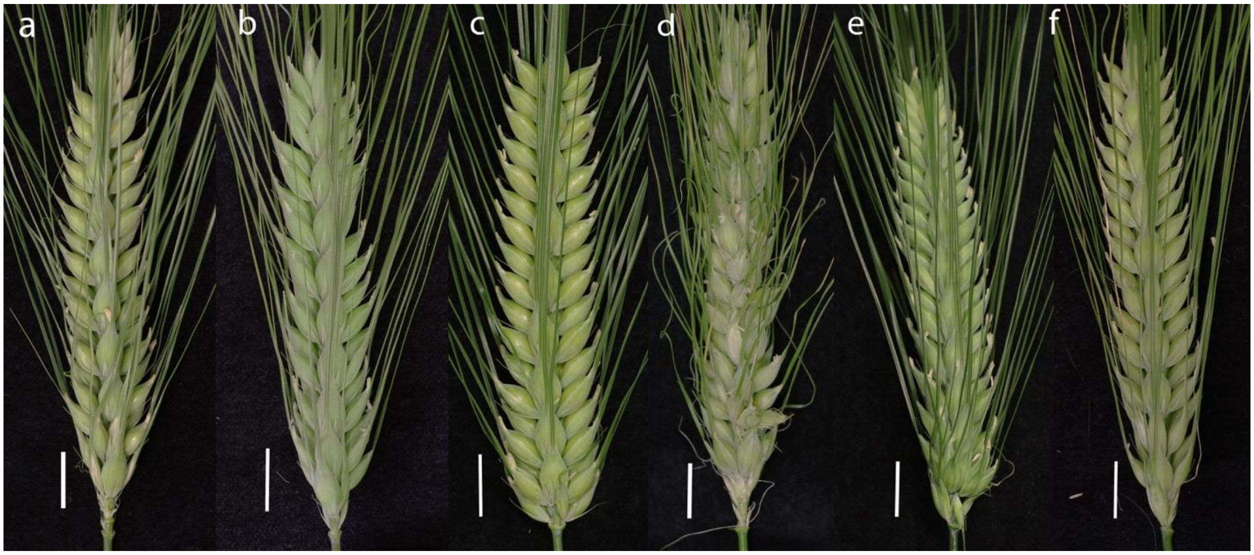

Fig. S4 Differential gene expression of meristem regulators in Bowman, *vrs3vrs4* and *vrs3vrs5* mutants. *RAMOSA-ENHANCER2* (*REL2*), *INDETERMINATE SPIKELET1* (*IDS1*), *VRS2* and *VRS4* transcript levels in developing spikes of *vrs3vrs4* and *vrs3vrs5* mutants and Bowman. Bar graphs show mean normalised expression (± SD) detected by qPCR (n=3). Significant differences to Bowman indicated by *, $P < 0.05$; **, $P < 0.01$; ***, $P < 0.001$ (student t-test). Bw, Bowman; mne, mean normalised expression. GP, glume primordium; LP, lemma primordium; SP, stamen primordium.

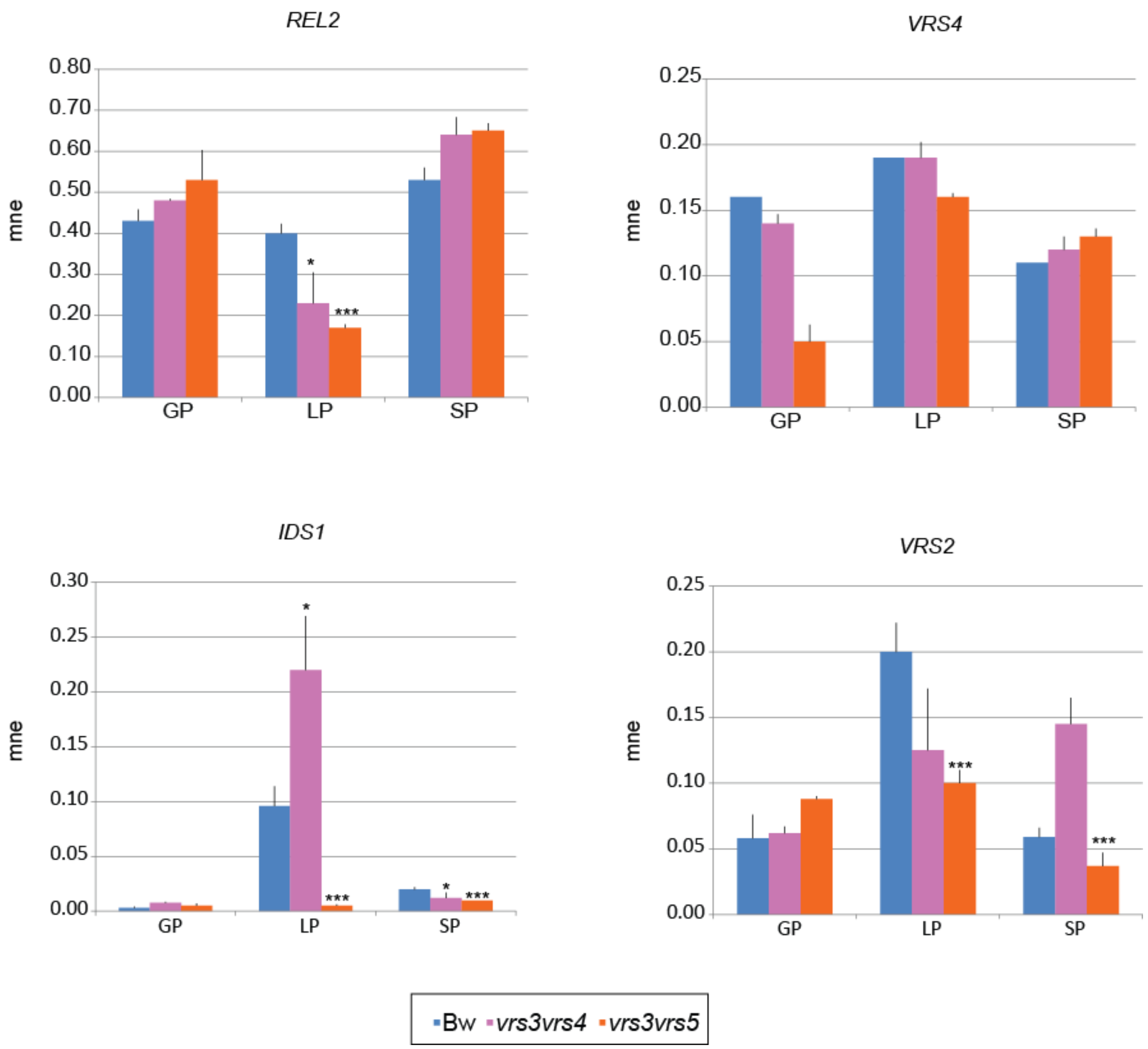

Fig. S5 Combined grain area from Bowman, single *vrs* mutants and double *vrs* mutants. Violin plots show grain area distribution from all grain including those from central spikelets, lateral spikelets, and (when present) additional spikelets. Grain harvested from the main culm and tallest tiller per individual. n = 10 individuals.

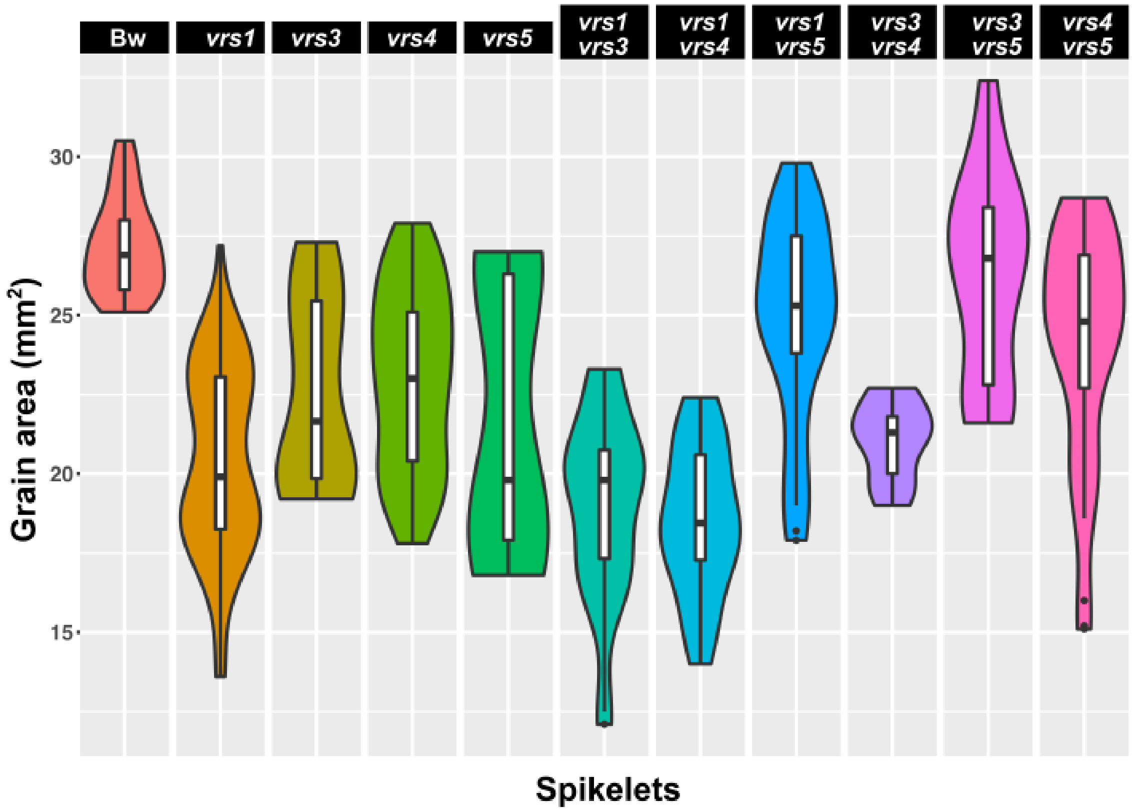

Fig. S6 Tiller outgrowth rate over time in Bowman and single *vrs* mutants and double *vrs* mutants. Graphs show duration and rate of tiller production in Bowman, single and double *vrs* mutants. Slope indicated by red lines showing rate of tiller production and goodness of fit indicated by R^2 correlation. Black circle symbols indicate window of active tiller outgrowth; dag, days after germination.

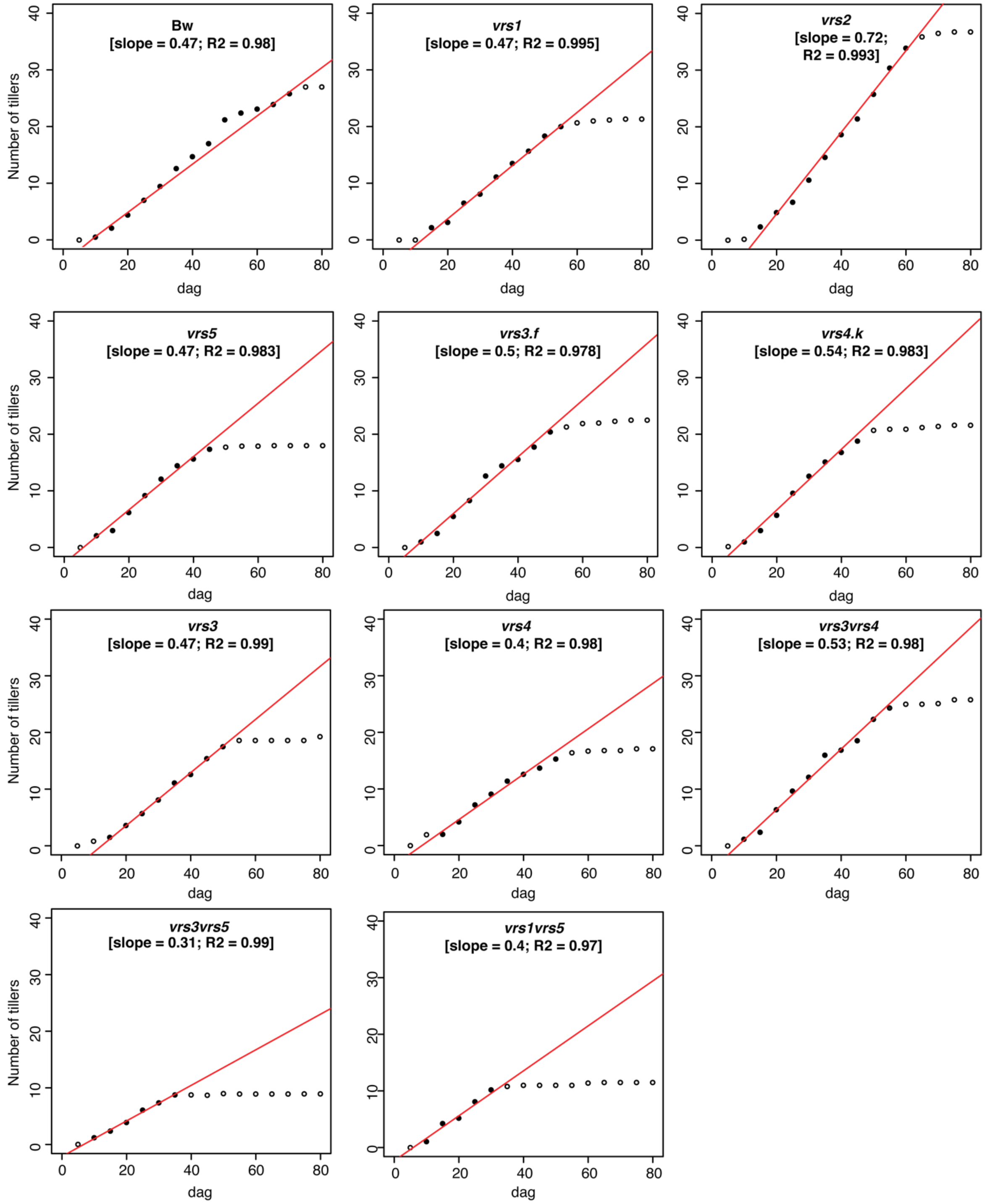

Fig. S7 Early tillering phase across Bowman and single *vrs* mutants (a) Tiller number was recorded at five days after germination (dag) and repeated at two to three day intervals until 23 dag (n=10 individuals/ genotype). Plants were grown in 7 cm pots under long day glasshouse conditions. Tiller number included tillers with visible spikes as well as 'vegetative tillers' without emerged spikes. Dots represent means (±SD). **(b)** Apex stage was determined at each time point in plants grown along side. VM, vegetative meristem; DR, double ridge; TSM, triple spikelet meristem; GP, glume primordium; LP, lemma primordium; SP, stamen primordium; AP, awn primordium; WA, white anther; GrA, green anther.

(a)

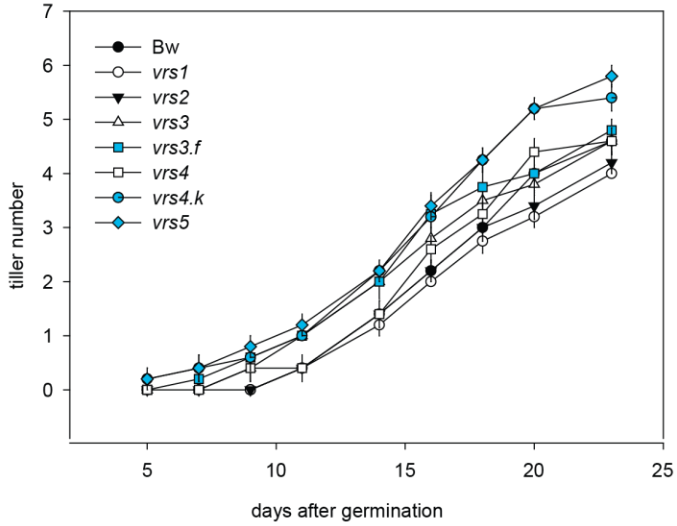

(b)

	5dag	7dag	9dag	11dag	14dag	16dag	18dag	20dag	23dag	35dag
**Bw**	VD	VD	early DR	DR	TSM	GP/LP	LP/SP	SP	AP	WA/GrA
vrs1	veg	VD	early DR	DR	TSM	GP/LP	LP/SP	SP	AP	WA/GrA
vrs2	VD	VD	VD	VD	early DR	DR/TSM	TSM	GP	LP/SP	WA/GrA
vrs3	VD	VD	early DR	DR	TSM	GP/LP	LP/SP	SP	AP	WA/GrA
vrs3.f	VD	VD	early DR	DR/TSM	TSM	GP/LP	LP/SP	SP	AP	WA/GrA
vrs4	VD	VD	early DR	DR	TSM	GP/LP	LP/SP	SP	AP	WA/GrA
vrs4.k	VD	VD	early DR	DR/TSM	TSM	GP/LP	LP/SP	SP	AP	WA/GrA
vrs5	VD	VD	DR	DR/TSM	TSM	GP/LP	LP/SP	SP	AP	WA/GrA

Table S6 Spikelet parameters in single *vrs* mutants and Bowman compared by ANOVA Values are mean (±SD) from awned spikelets from n = 10 spikes per line. Means with the same letters are not statistically different from each other by ANOVA (p<0.05).

Line	rachis nodes	spikelets/ spike (/ node)	spikelets setting grain/ spike (/ node)	empty awned spikelets/ spike (/ node)	grain area (mm²)^	TGW (g)	Final tiller number
**Bw**	22.4 ±1.3 b	66.9 ±3.4b (3.0 ±0.0)b	19.1 ±2.5 a (0.9 ±0.1)d	3.0 ±1.5 a (0.1 ±0.1)d	27.2 ±1.5 b	56.5 ±4.6 c	27 ±3.2 c
vrs1	20.4 ±0.5 a	60.3 ±2.1bc (3.0 ±0.1)b	52.7 ±4.2 c (2.6 ±0.2)c	7.7 ±4.2 b (0.4 ±0.2)c	22.2 ±3.1 a	37.7 ±11 ab	21.3±2.2 ab
vrs2	**23.6 ±0.5 b**	76.3 ±3.1ab (3.1 ±0.2)b	14.6 ±2.9 a (0.6 ±0.1)a	20 ±1.4 d (0.8 ±0.0)a	24.2 ±3.1 ab	48.1 ±7.6 bc	36.8±2.8d
vrs3.f	20.6 ±0.5 a	62.5 ±1.6bc (3.0±0.1)b	35.8 ±7.4 b (1.7 ±0.4)b	8.75 ±4.3 bc (0.4 ±0.2)cd	23.2 ±4.1 a	39.6 ±13 ab	22.5±2.6 b
vrs4.k	19.6 ±1.1 a	78.4 ±6.6a (4.0±0.3)a	56.4 ±4.9 c (2.9 ±0.2)c	13.4 ±4.6 c (0.7 ±0.3)b	20.7 ±1.7 a	34.9 ±6.5 a	21.6±2.1b
vrs5	19.5 ±1.6 a	58.5 ±4.9c (3±0.1)b	34.5 ±3.2 b (1.8 ±0.3)b	8.5 ±4.4 abc (0.4 ±0.2)cd	22.3 ±4.4 a	43.1 ±16 ab	18 ±1.4a

^Averaged values for grains deriving from central, lateral and additional spikelets. Bw, Bowman

Methods S1 Supplemental methods for genotyping and qPCR

(1) KASP Genotyping. Each reaction contained 1 µl of 20 ng genomic DNA, 3 µl H2O, 4 µl 2x KASP master mix (LGC Genomics) and 0.11 µl allele-specific primers (LGC Genomics, listed below). Reactions were performed according to the following steps: 2 min at 20°C; 15 min at 94°C; 10 touch-down cycles of 20 sec at 94°C, 1 min at 62°C, with -0.7°C per cycle; 32 cycles of 20 s at 94°C, 1 min at 55°C, 2 min at 20°C. All assays were run on an ABI 3700 Step-One Plus Real Time PCR machine (Applied Biosystems). (2) VRS1 Genotyping. PCR amplicons (primers listed below) were purified with ExoSAP-IT™ PCR Product Cleanup Reagent (ThermoFisher Scientific) and sequenced using the BigDye Terminator version 3.1 Ready Reaction Cycle Sequencing Kit (Applied Biosystems). Samples were sequenced on an ABI3730 and trimmed sequences were analyzed with Sequencher 5.2.3 software (GeneCodes). (3) Quantitative RT-PCR (qRT-PCR). The qRT-PCR for each gene was run in three (technical and biological) replicates and expression measured using TaqMan technology (Roche). Relative expression levels were calculated using two stable expressed internal reference genes, HvACTIN2 and PROTEIN PHOSPHATASE 2 (HvPP2A). Sequences of gene-specific oligonucleotides (listed below) and probe numbers were designed using Roche Universal Probe Library Design Center website.

Primer pair efficiency was tested by standard curve using a 1:5 dilution series over 5 dilution points. The qRT-PCR reaction was performed in 25 µl volume consisting of 2x Universal probe library master mix + ROX Fast Start TaqMan (Roche), 10 µM forward and reverse primer, 10 µM Universal probe library hydrolysis probe, and run in a 96-well plate on a ABI 3700 Step-One Plus Real Time PCR machine (Applied Biosystems) with the following conditions: 10 min at 95°C, 40 cycles of 15 s at 95°C, 1 min at 60°C using the comparative CT program. Statistical differences were assessed with a Student's t-test (two-tailed).

Primers

Kasp Genotyping

gene	mutant line (allele)	Polymor-phism	FAM allele	VIC (Hex) allele	DNA sequence designed to
HvVRS3	BW419 (*int-a.1; vrs3*)	GC/--	*Vrs3.x*	*int-a.1*	TCTGCCTTCATCATGGTAGGAAATCCTGACATTGCTGTTTCTCTAGCCTTCCCC TGAAGTTTGTAATTTATCTGATTGTTTGTTGATTGGAGTACAGAACAAGAACT[GC/-]GGAGCTGCTCTTGTAAATCTGACCGGATTGTCTACGTCAGAGAAGACAT ACTGGAGTTAGAGGCTATATATAGAAAATTCGAGCAGGATATTCGCCTGGAT AAGGAAACAAGTGCTAATATCTCGTATAAGCAAGCTGCGATTTCTGATATTGG TGTCGATCATGGTCCATCAGTTGGCA
HvVRS4	BW423 (*int-e.58; vrs4*)	T/C	*Vrs4*	*int-e.58*	TTCCCGCCGGAGGAGCCGCAGAAGTTCGCCAACGTGCACAAGGTGTTCGGCG CCAGCAACGTGACCAAGCTGCTCAACGAGCTGCCGCCGCACCAGCGGGAAGA CG[T/C]CGTGAGCTCGCTGGCCTACGAGGCGGAGGCGCGGGTCAAGGACCCC GTCTACGGCTGCGTCGGCGCCATCTCCGTGCTCCAGCGCCAGGTCCACCGCCT CCAGAAGGAGCTCGACGCCGCGCACACCGAGCTCCTCCGGTACGCCTGCGGC GAGCTCGGCAGCATCCCCACCGCGCTCCCCGTTGTCACGGCCGG
HvVRS5	BW421 (*int-c.5*)	A/-	*Int-c.b*	*int-c.5*	CCCCGACAAGGAGTCGAGGACAAAGGCGAGGGAGAGGGCAAGGGAGCGGA CGAGGGAGAAGAACCGGATGCGATGGGTGACGCTCGCGTCCACAATCAACA TCGAGCCGGCAACCACCGGCATGGCGGCGGCGAGGCTGGACGAGTTGGTCA CCAGCCCCAACAATTTGATCAATCGCTCCTCGTCCATGAACACGCCAGGCGCT GA[A/-]TTGGAGGAGGGGTGCTCGTCGTCCATGCCGAGCGAAGCGATCATGGC TGGCTTCGGCAATGGAGGGTACGGCAGCATCGGCAACTACTACCAGCACCAG CTGGAGCAGCAATGGGAGCTCGGTGGAGTGGTGTTTGCCAACTCCCAGCACT ACTGAGAAGCGACGACGTGCGTACACGGTACAAGAACTACTT

***VRS1* genotyping**

Gene	mutant line (allele)	Primer Pair (5'-> 3')	expected fragment size
HvVRS1	BW898 (*vrs1.a*)	Forward ACAGGCAACAGAACAACCTACC<br>Reverse CAAGAACGGAGAGGAACTGC	696 bp

qPCR primers					
**Gene**	**accession number**	**Primer Pairs (5'-->3')**	**UPL #**	**Efficiency (%)**	**R^2**
HvACT2	HORVU1Hr1G002840.4	Forward GCGAGTTGTCTGGGTCTTCT<br>Reverse ACATGGCAAGGACTTGAGAAA	129	97.5	0.999
HvPP2A	HORVU5Hr1G109430.2	Forward CGTCGCATCATGATCAAAGT<br>Reverse CGAGGTGAGTAACACGATGG	11	99.7	0.998
HvVRS1	HORVU2Hr1G092290.6	Forward CCCATAAAATAGCCGAGATAGC<br>Reverse AGGTTTCTGCCGATCTTGAA	70	98.0	0.969
HvVRS2	HORVU5Hr1G081450.1	Forward CAACATCGTCGTGTCATCG<br>Reverse GGGAACGAGCCGTAGAGC	9	98.6	0.929
HvVRS3	HORVU1Hr1G051010.5	Forward CACTTTCTTTATGAGTGGACGAAA<br>Reverse CAGAAGAGATTTCACGCCAGA	101	106	0.975
HvVRS4	HORVU3Hr1G016690.1	Forward GTGAACGCCATTAGCACCAT<br>Reverse GTGATCCATCCCAATGCTCT	77	99.0	0.999
HvVRS5	HORVU4Hr1G007040.1	Forward ACCATTCCTCCCCTCCATT<br>Reverse GCACCGGCACCGGCACAGAGGTAG	31	95.2	0.997
HvIDS1	HORVU5Hr1G112440.1	Forward GACTGCGGTGCCATGACTA<br>Reverse CAGAAAAAGCCCAACGGTTA	9	100	0.974
HvREL2	HORVU0Hr1G008690.1	Forward AAGACGATGACAACGTTTATGC<br>Reverse AGGATGGAATGCGAGAAAAG	77	95.6	0.993
BM3	HORVU0Hr1G003020.3	Forward CGAGGATATACCTATGGGCTGA<br>Reverse CGGACTTTGACCAATGGACT	164	99.5	0.929
BM8	HORVU2Hr1G063800.7	Forward TCTCGGGCGATGTGTCTACT<br>Reverse AAGGTTGCATGTTGGATGGT	156	106	0.975
HvLOG1	HORVU5Hr1G124750.1	Forward CGCGTCCTCCTTTATGAATC<br>Reverse AACTTCTTCCTGTCCTTCATCG	76	92.7	0.955
HvCKX2	HORVU3Hr1G027460.1	Forward TGTCCGATGGTGATCTTGG<br>Reverse TCCTCGCAAAACCGTAAGAT	25	99.5	0.996
